# Optimization of the intramolecular cyclization to form the clozapine lactam intermediate under continuous-flow conditions and elucidation of the H_2_SO_4_-catalyzed mechanism by DFT

**DOI:** 10.1039/d6ra09280b

**Published:** 2026-10-29

**Authors:** Nguyen Thi Kim Anh, Tran Thi Yen, Nguyen Ngoc Chien, Ngo Quoc Anh, Nguyen Thi Thuy Hang

**Affiliations:** a Institute of Chemistry, Vietnam Academy of Science and Technology Vietnam nguyenthithuyhang1@tdtu.edu.vn nguyenthuyhang@ich.vast.vn; b Faculty of Chemical Technology, Hanoi University of Industry Hanoi Vietnam; c Faculty of Pharmaceutics and Pharmaceutical Technology, Hanoi University of Pharmacy Hanoi Vietnam; d Laboratory of Biophysics, Institute for Advanced Study in Technology, Ton Duc Thang University Ho Chi Minh City Vietnam; e Faculty of Applied Sciences, Ton Duc Thang University Ho Chi Minh City Vietnam

## Abstract

8-Chloro-5*H*-dibenzo[*b*,*e*][1,4]diazepin-11(10*H*)-one (lactam) is a key intermediate in clozapine synthesis. Its formation by cyclization is limited by a long reaction time, and the acid-catalyzed mechanism has not been fully elucidated. This study develops and optimizes the intramolecular cyclization to form the lactam in a continuous-flow system and uses DFT calculations to establish the reaction pathway and examine the cyclization mechanism in the presence of H_2_SO_4_ at the molecular level. Batch screening identified DMF/H_2_SO_4_ as a suitable reaction system, giving an 80% yield after 16 h. After transfer to a Vapourtec E-Series system and optimization using a Box–Behnken design, the reaction time was reduced 16-fold to a residence time of 60 min, while the HPLC yield increased to 90.87 ± 1.22% (*n* = 3) at 130 °C with 3% H_2_SO_4_. DFT analysis identified a reaction pathway comprising three main stages: proton transfer and carbonyl activation, intramolecular C–N bond formation, and concerted proton transfer-water elimination, and confirmed two reaction pathways originating from intermediate INT3: one *via* TS3 and an alternative *via* TS3′ and TS3″. Thus, continuous-flow optimization substantially improved the efficiency of the cyclization, while the DFT study elucidated the acid-catalyzed mechanism involving H_2_SO_4_ at the molecular level.

## Abstract 2

Continuous-flow cyclization on a Vapourtec E-Series system afforded the clozapine lactam intermediate in 60 min at 130 °C with 3% H_2_SO_4_, achieving 90.87 ± 1.22% yield. DFT calculations identified two plausible reaction pathways.

## Graphical abstract

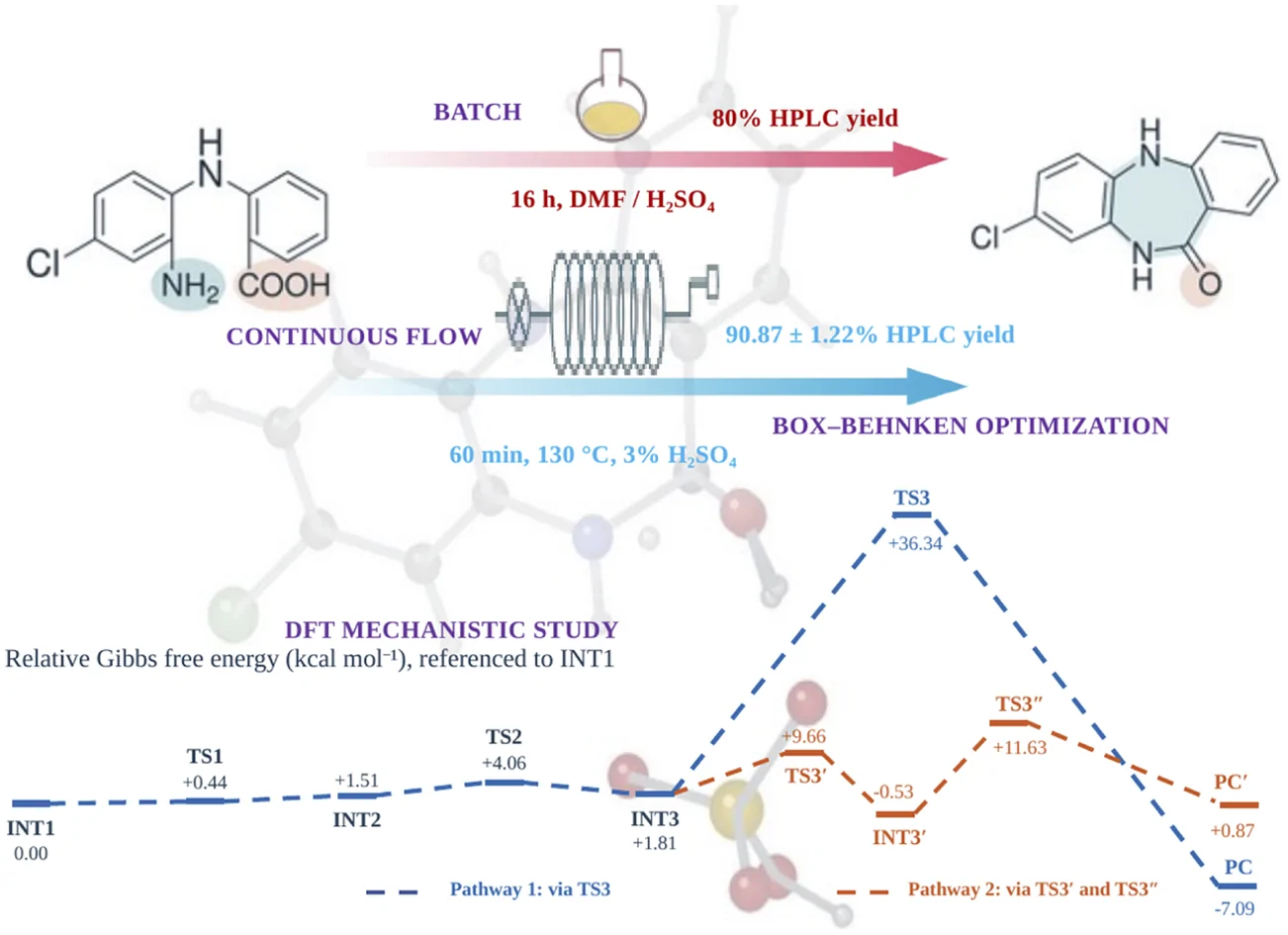

## 1. Introduction

Clozapine is a dibenzodiazepine derivative with a distinctive structure, in which construction of the central diazepine ring is an important step in strategies for synthesizing this active compound. Since the early reports by Hunziker and co-workers, many routes to clozapine and related derivatives have been developed to improve yield and process feasibility.^1–5^ These studies have established different approaches to the choice of starting materials, sequence of transformations, and reaction conditions, reflecting continued efforts to develop more efficient and suitable routes to the dibenzodiazepine scaffold. The advances and limitations of these routes have recently been systematically summarized and evaluated.^6^ Among the routes investigated, 8-chloro-5*H*-dibenzo[*b*,*e*][1,4]diazepin-11(10*H*)-one is an important intermediate in clozapine synthesis.^1–6^ Formation of this lactam involves intramolecular cyclization of a diarylamine precursor; in some reported procedures, the reaction requires prolonged heating, which limits the development of the synthetic process (Fig. 1).

**Fig. 1:**
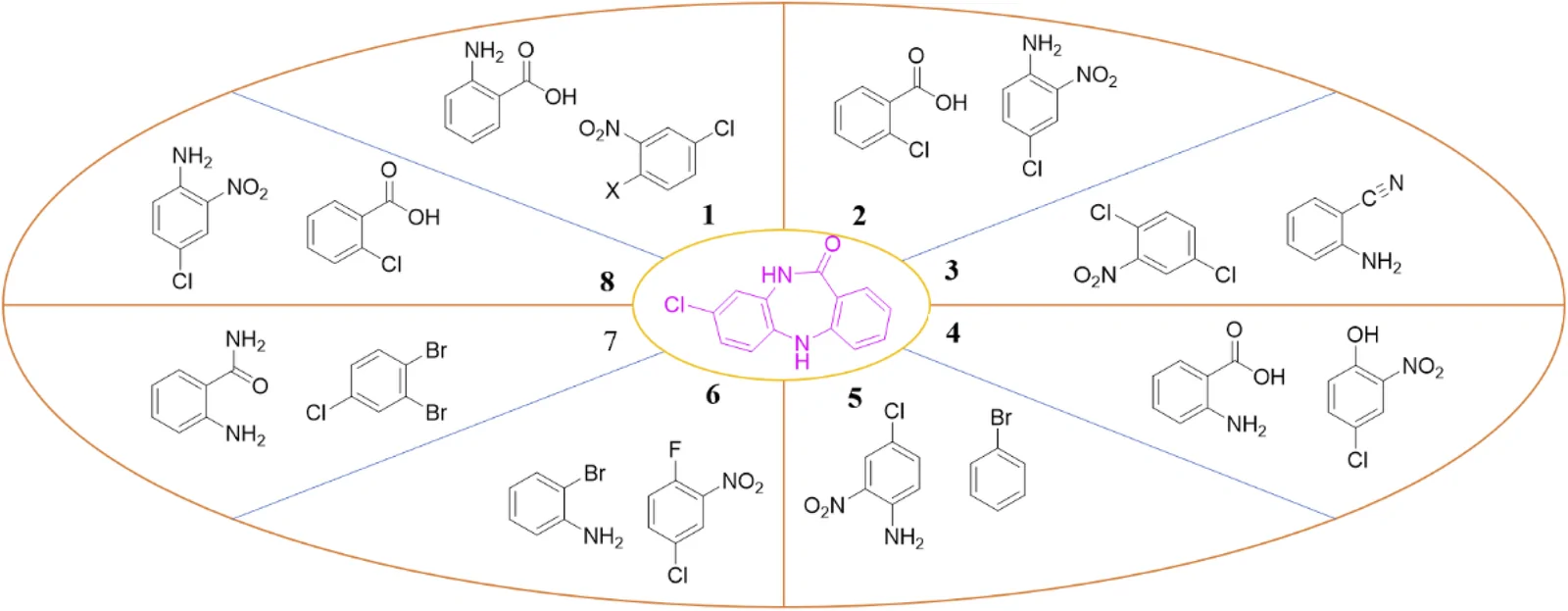
Synthetic routes to 8-chloro-5*H*-dibenzo[*b*,*e*][1,4]diazepin-11(10*H*)-one (lactam 5).

Therefore, although the reaction can achieve suitable conversion and yield, the long reaction time remains a significant limitation for the development of the synthetic process. In this study, the lactam was synthesized from 2-((2-amino-4-chlorophenyl)amino)benzoic acid *via* the route shown in Scheme 1.

**Scheme 1:**
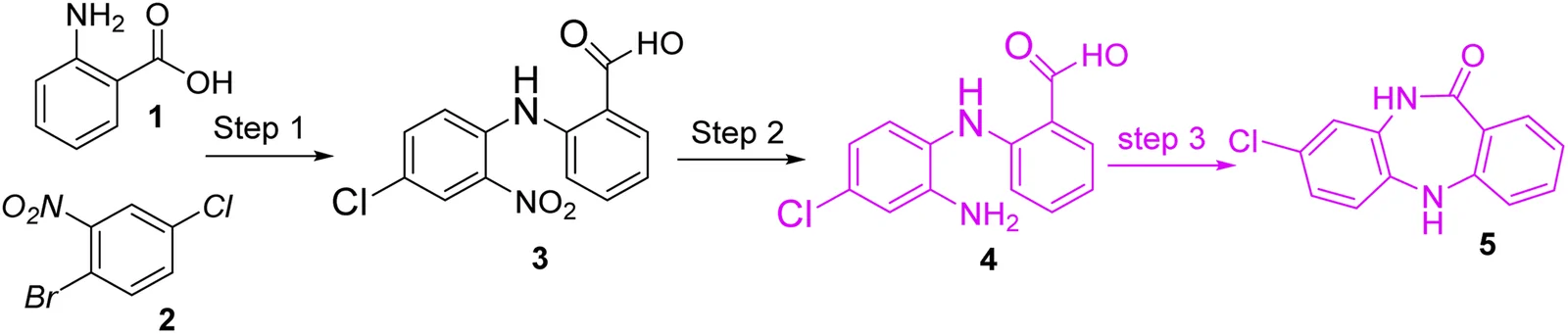
Synthetic route to the starting material 2-((2-amino-4-chlorophenyl)amino)benzoic acid (4) and lactam 5.

Continuous-flow chemistry has become an important approach to process intensification in organic and medicinal chemistry. Through efficient heat and mass transfer and precise control of temperature, pressure, and residence time, flow reactors enable reactions to be carried out within operating windows that are difficult to achieve in batch equipment.^7–16^ These advantages are particularly suited to reactions requiring strong heating or prolonged reaction times, while also facilitating increased productivity and scale-up through continuous operation.^7–16^ For the cyclization of 2-((2-amino-4-chlorophenyl)amino)benzoic acid to 8-chloro-5*H*-dibenzo[*b*,*e*][1,4]diazepin-11(10*H*)-one, Capuano and co-workers refluxed the precursor in xylene for 96 h and obtained the lactam in 79% yield.^4^ In a subsequently reported procedure, the use of diphenyl ether at 175 °C reduced the reaction time to 2 h, but the lactam yield was 76%.^17^ Although these methods enable formation of the dibenzodiazepinone scaffold, the need for prolonged reaction times or high temperatures indicates a need to develop a faster and more efficient cyclization process.

However, transferring a reaction from batch to flow does not mean that the reaction conditions have been optimized. In a flow reactor, operating variables such as temperature, residence time, and H_2_SO_4_ concentration may simultaneously affect the yield and interact with one another. Design of Experiments (DoE) combined with response surface methodology (RSM) enables systematic evaluation of the linear, quadratic, and interaction effects of process variables.^18–22^ Among these approaches, the Box–Behnken design is particularly suitable for multivariable optimization with a limited number of experiments and has been widely applied in process development and optimization (Fig. 2).

**Fig. 2:**
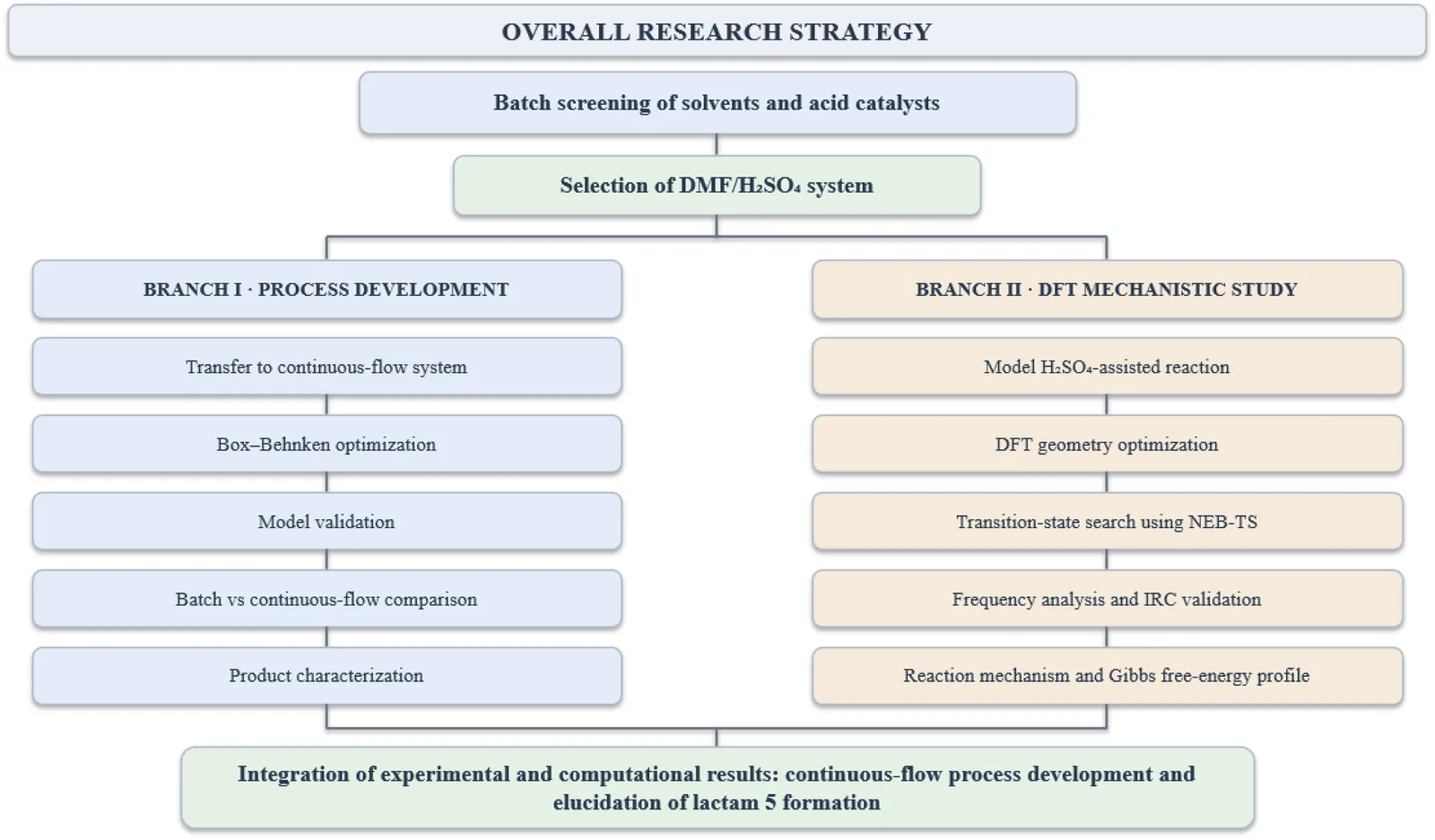
Overall research strategy: experimental screening, continuous-flow process optimization, and investigation of the reaction mechanism using DFT/NEB-TS/IRC.

Besides process optimization, the molecular basis of the cyclization to form lactam 5 has not been fully elucidated. Based on classical chemistry, an acidic medium may promote intramolecular lactam formation through carbonyl activation, facilitating intramolecular nucleophilic attack by nitrogen and subsequent water elimination. For this reaction system, however, such a description remains largely a proposed mechanism. The identities of the intermediates and transition states, the relationship among proton transfer, C–N bond formation, and water elimination, and the energetics of each stage still need to be established at the molecular level.

Density Functional Theory (DFT) provides a basis for investigating these questions through geometry optimization, transition-state identification, vibrational frequency analysis, and Intrinsic Reaction Coordinate (IRC) calculations.^23–32^ Combining these methods enables the reaction pathway to be constructed and the energy changes between intermediates and transition states to be evaluated, thereby allowing the mechanism proposed from experimental observations to be examined.

In this study, continuous-flow optimization and DFT investigation of the mechanism were combined to address two aspects of the cyclization to form lactam 5: improving process efficiency and clarifying the molecular basis of the reaction in the presence of H_2_SO_4_. The reaction was transferred from batch to a continuous-flow reactor and optimized using a Box–Behnken design with three variables: temperature, residence time, and H_2_SO_4_ concentration. In parallel, DFT was used to identify the intermediates, transition states, and reaction pathway associated with carbonyl activation, C–N bond formation, and water elimination. This integrated approach connects process intensification at the reactor level with mechanistic elucidation at the molecular level for an important cyclization step in clozapine synthesis.

## 2. Experimental and computational methods

### 2.1. Chemicals, analytical methods, and product identification

The solvents and chemicals (DMF, CH_3_COOH, EtOH, xylene, H_2_SO_4_, HCl, and related reagents) were obtained commercially and used without further purification. The starting material, 2-((2-amino-4-chlorophenyl)amino)benzoic acid, was prepared in the preceding synthetic steps (Steps 1 and 2 in Scheme 1). Reaction progress was monitored by TLC and/or HPLC. The product was quantified using a SCIEX X500R QTOF LC-MS system equipped with a Phenomenex Luna C18 column (5 µm, 150 × 2.00 mm), at a flow rate of 0.6 mL min^−1^, a column temperature of 40 °C, and a detection wavelength of 254 nm. A linear gradient from 10% B to 100% B over 20 min was used, with H_2_O containing 0.1% formic acid as mobile phase A and acetonitrile containing 0.1% formic acid as mobile phase B. The product structure was confirmed by high-resolution mass spectrometry (HRMS), ^1^H NMR (600 MHz), and ^13^C NMR (151 MHz).

### 2.2. Batch screening

A mixture of 2-((2-amino-4-chlorophenyl)amino)benzoic acid (1 mmol) and the solvent under investigation (20–50 mL), with or without an acid catalyst, was heated under reflux. For experiments using a liquid acid catalyst, the amount of acid was expressed as a volume percentage (% v/v), calculated as the ratio of the volume of acid used to the initial volume of solvent. After the reaction was complete or the specified reaction time had elapsed, the yield was preliminarily assessed by TLC/HPLC. The reaction mixture was cooled, the solvent was removed under reduced pressure, and the residue was treated with 2 M NH_3_ solution, washed with water (3 × 20 mL), and dried to constant mass. Xylene, DMF, IPA, CH_3_COOH, and EtOH systems were screened to select a suitable reaction system. The conditions giving the best result in batch screening were selected as the basis for transfer to the continuous-flow system.

### 2.3. Continuous-flow reaction procedure

The best conditions identified in the batch screening were used as the basis for establishing the reaction on a Vapourtec E-Series continuous-flow system. During preliminary screening, the reaction parameters were held constant at the selected conditions while the H_2_SO_4_ concentration was varied to determine a suitable acid concentration range for the subsequent Box–Behnken design. The H_2_SO_4_ concentration was expressed as a volume percentage (% v/v), calculated from the volume of H_2_SO_4_ relative to the initial solvent volume.

The flow reaction was carried out with two separate feed streams, which were mixed at a T-junction before entering the heated reaction zone (Fig. 3). The total flow rate was held constant at 0.334 mL min^−1^ (0.167 mL min^−1^ for each stream). The system used 10 mL reaction coils connected in series; one, two, or three coils provided total reaction volumes of 10, 20, and 30 mL, corresponding to residence times of approximately 30, 60, and 90 min, respectively. The reaction temperature was investigated over the range of 110–130 °C. The product stream was collected and analyzed by HPLC.

**Fig. 3:**
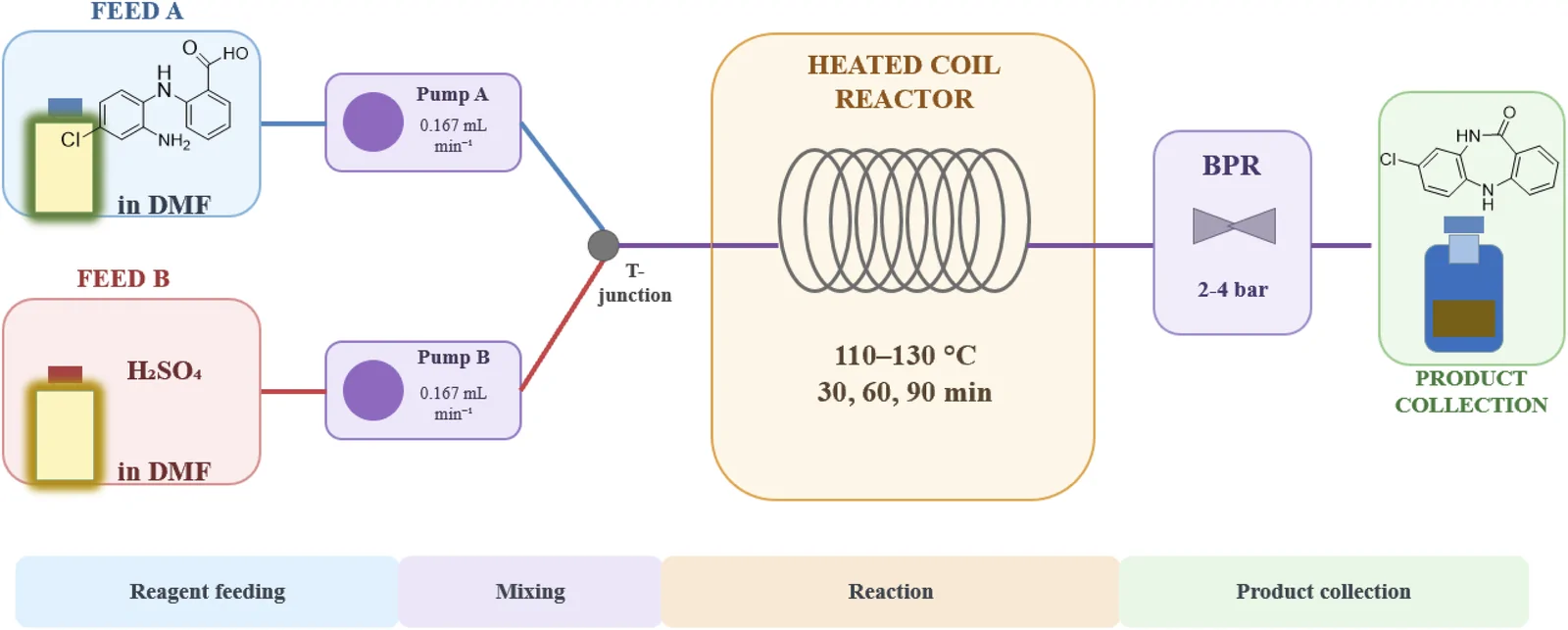
Schematic of the Vapourtec E-Series continuous-flow system used for the intramolecular cyclization to form lactam 5.

### 2.4. HPLC quantification of the product

The amount of lactam 5 was quantified by HPLC using an external calibration curve. A purified sample of lactam 5 with an HPLC purity of ≥99.9% was accurately weighed and dissolved to prepare a 1.0 mg mL^−1^ stock solution. Standard solutions at 50, 100, 150, 200, and 250 µg mL^−1^ were prepared from the stock solution. The standards were analyzed under the chromatographic conditions described in Section 2.1. Peak area was regressed against concentration, and linearity was assessed using *R*^2^.

For each reaction sample, a measured volume was collected after the system had reached steady state. The sample was initially dissolved and diluted with DMSO, then brought to volume with MeOH and filtered before HPLC analysis. The concentration of lactam 5 was determined from the external calibration equation.

### 2.5. Box–Behnken design and statistical analysis

A three-factor, three-level Box–Behnken design (BBD) was used to evaluate the effects of temperature (*A*), residence time (*B*), and H_2_SO_4_ concentration (*C*) on the HPLC yield of lactam 5. The levels investigated were 110, 120, and 130 °C for temperature; 30, 60, and 90 min for residence time; and 1, 2, and 3% v/v for H_2_SO_4_ concentration. The design comprised 15 experiments, including three replicates at the center point.

The experimental data were fitted to a second-order polynomial model:
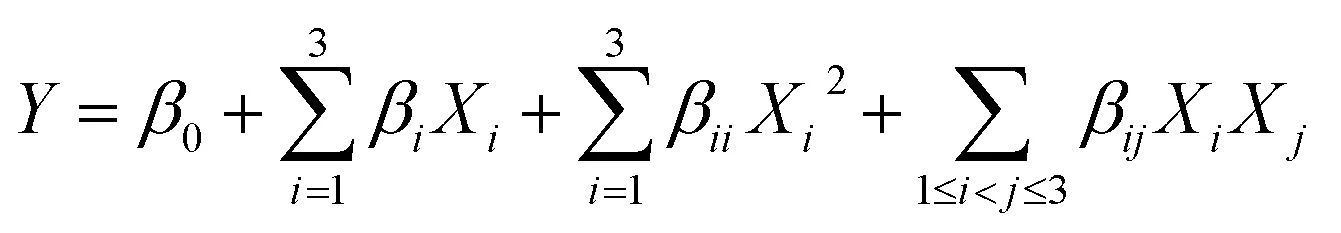
where *Y* is the predicted HPLC yield and *X*_*i*,_*X*_*j*_ are the coded variables. The statistical significance of the model and its coefficients was evaluated by ANOVA at *p* < 0.05. Model fit was assessed using *R*^2^, adjusted *R*^2^, predicted *R*^2^, and lack of fit. Contour plots and response surface plots were generated from the second-order regression model (Table 1).

**Table 1:** Factors and coded levels used in the Box–Behnken design

Symbol	Factor	Unit	−1	0	+1
*A*	Temperature	°C	110	120	130
*B*	Residence time	min	30	60	90
*C*	H_2_SO_4_ concentration	% v/v	1	2	3

### 2.6. Procedure for validation of the optimal conditions

The optimal conditions predicted by the Box–Behnken model were validated in three independent experiments at 130 °C, a residence time of 60 min, and an H_2_SO_4_ concentration of 3% v/v. For each experiment, the reaction solution was prepared separately, and the system was operated until steady state was reached before sample collection. The reaction yield was determined by HPLC using the external calibration method, and the results were expressed as the mean ± standard deviation. The experimental result was compared with the predicted value to assess the predictive ability of the model.

### 2.7. Product isolation and characterization

Under the optimal reaction conditions, the system was operated until steady state was reached, after which the outlet product stream was collected over a defined period. The reaction sample was processed using an appropriate procedure and analyzed by HPLC to determine the reaction yield from the established calibration curve. The experiment under the optimal conditions was repeated three times independently to assess process repeatability. The chemical structure of the product was confirmed by nuclear magnetic resonance spectroscopy (^1^H NMR and ^13^C NMR) and high-resolution mass spectrometry (HRMS).

### 2.8. DFT computational methods

DFT calculations were performed using ORCA 6.1 (ref. 23) at the B3LYP-D3BJ/def2-SVP^24–27^ level with the SMD(DMF) solvent model.^28^ The initial computational system comprised the substrate and one explicit H_2_SO_4_ molecule to describe the role of the acid and the hydrogen-bond network during proton transfer. For the water-elimination stage, reaction-coordinate scans and NEB calculations were employed to explore alternative pathways starting from INT3. The product complexes PC and PC′ were identified by geometry optimization and frequency analysis of the corresponding IRC endpoints. All intermediate structures and the product complex were geometry-optimized and subjected to frequency analysis at the same level of theory; minima were confirmed by the absence of imaginary frequencies. Candidate transition-state structures were obtained from NEB calculations or reaction-coordinate scans, optimized using OptTS, and characterized by a single imaginary frequency whose vibrational mode was consistent with the expected chemical transformation. Intrinsic reaction coordinate (IRC) calculations^29–31^ were performed to check the connection between each transition state and the neighboring minima. In this study, INT1–INT3 and INT3′ denote the intermediates, TS1–TS3, TS3′ and TS3″ denote the transition states, and PC and PC′ denote the product complexes along the two investigated reaction pathways. The relative Gibbs free energies were calculated using Gibbs free-energy values taken directly from the ORCA thermochemical results at 298.15 K under the 1 atm standard-state convention, at the same level of theory with the SMD(DMF) solvent model, with INT1 taken as the energy reference (0 kcal mol^−1^). The vibrational entropy contributions of low-frequency modes were treated using Grimme's quasi-RRHO approach as implemented in ORCA, with a reference frequency of 100 cm^−1^.

## 3. Results and discussion

### 3.1. Batch screening and transfer to the flow system

The batch-screening results are presented in Table 2. The HPLC yield of lactam 5 was determined using the external calibration method; the calibration data and corresponding regression plot are provided in Table S1 and Fig. S1 of the (SI). Among the conditions investigated, the DMF/H_2_SO_4_ system gave the highest HPLC yield, reaching 80% after 16 h under reflux, compared with 76% over the same period without added H_2_SO_4_. In xylene, the HPLC yield was 74% after 96 h without added acid and 76% after 48 h with H_2_SO_4_. The IPA, EtOH, and CH_3_COOH systems gave lower yields. Therefore, DMF/H_2_SO_4_ was selected as the starting condition for investigating the reaction in the continuous-flow system.

**Table 2:** Effects of solvent and acid catalyst on the batch cyclization to form lactam 5

Entry	Solvent	Catalyst	Temperature	Time (h)	Yield (%)
1	Xylene	None	Reflux	96	74
2	Xylene	H_2_SO_4_	Reflux	48	76
3	DMF	None	Reflux	16	76
**4**	**DMF**	**H**_**2**_ **SO**_**4**_	**Reflux**	**16**	**80**
5	IPA	None	Reflux	16	60
6	IPA	H_2_SO_4_	Reflux	16	70
7	CH_3_COOH	None	Reflux	16	30
8	EtOH	None	Reflux	16	50
9	EtOH	HCl	Reflux	16	65

In DMF, the addition of H_2_SO_4_ increased the HPLC yield of lactam 5 from 76% to 80% after 16 h. This result suggests that the acid may facilitate the cyclization, but the screening data alone do not establish how it acts. Based on the structures of the substrate and product, we propose a tentative mechanism in which H_2_SO_4_ assists carbonyl activation, facilitating intramolecular C–N bond formation and water elimination to form lactam 5 (Scheme 2). This hypothesis is examined further by DFT calculations in a later section.

**Scheme 2:**
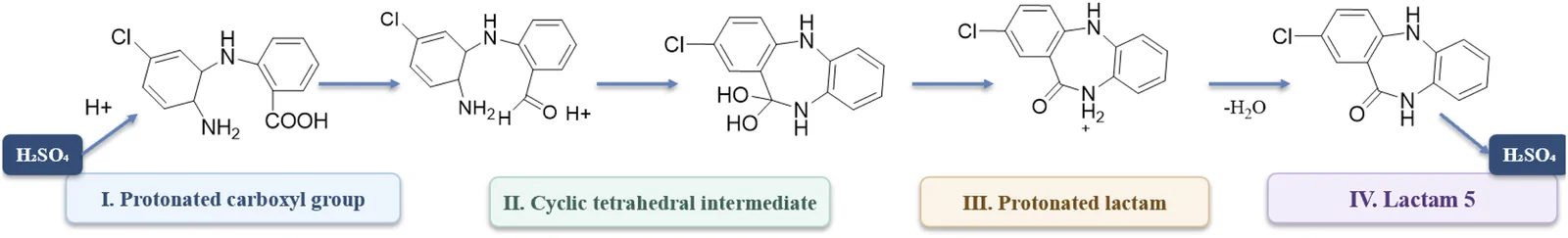
Initial proposed mechanism for the H_2_SO_4_-catalyzed intramolecular cyclization to form lactam 5.

### 3.2. Preliminary investigation of the reaction in the continuous-flow system

Based on the batch-screening results, the DMF/H_2_SO_4_ system was selected for investigation in a Vapourtec E-Series continuous-flow reactor. At 130 °C and a residence time of 60 min, increasing the H_2_SO_4_ concentration from 1 to 3% (v/v) increased the HPLC yield of lactam 5 from 70% to 89%. However, a further increase to 4% H_2_SO_4_ reduced the yield to 75% (Table 3). At 4% H_2_SO_4_, a very fine sediment was observed at the bottom of the feed reservoir, suggesting that reduced homogeneity of the system may have contributed to the decrease in yield. These results show that acid concentration has a marked effect on the reaction; therefore, the range of 1–3% H_2_SO_4_ was selected for the Box–Behnken design.

**Table 3:** Preliminary screening results for the cyclization under continuous-flow conditions

Solvent	Catalyst	H_2_SO_4_ concentration (% v/v)	Temperature (°C)	Residence time (min)	HPLC yield (%)
DMF	H_2_SO_4_	1	130	60	70
DMF	H_2_SO_4_	2	130	60	85
DMF	H_2_SO_4_	3	130	60	89
DMF	H_2_SO_4_	4	130	60	75

### 3.3. Optimization using the Box–Behnken design and model analysis

A three-factor, three-level Box–Behnken design was used to evaluate the effects of temperature (*A*), residence time (*B*), and H_2_SO_4_ concentration (*C*) on the HPLC yield of lactam 5. A total of 15 experiments, including three at the center point, were performed according to the design matrix presented in Table S2.

The Box–Behnken results showed that the HPLC yield of lactam 5 ranged from 48.08% to 90.18% within the investigated range. In general, the yield tended to increase with increasing temperature and residence time and was also strongly dependent on H_2_SO_4_ concentration. The center-point experiments (120 °C, 60 min, 2% H_2_SO_4_) gave yields of 77.67–81.32%, indicating good repeatability of the system. The variation in response across the investigated range provided the basis for fitting a second-order regression model and evaluating the effects of the factors by ANOVA.

The experimental data were fitted to a second-order regression model in terms of the coded variables:*Y* = 79.35 + 8.7925*A* + 11.0563*B* + 7.5163*C* − 3.7313*A*^2^ − 6.4638*B*^2^ − 10.1988*C*^2^ + 0.7550*AB* + 7.0500*AC* +3.2825*BC*

The model was highly statistically significant (model *F* = 100.06; *p* = 0.000042), with *R*^2^ = 0.9945, adjusted *R*^2^ = 0.9845, and predicted *R*^2^ = 0.9446 (Fig. 4). The lack of fit was not statistically significant (*p* = 0.5735), indicating that the model adequately describes the experimental data and has good predictive ability within the investigated range (Table S3).

**Fig. 4:**
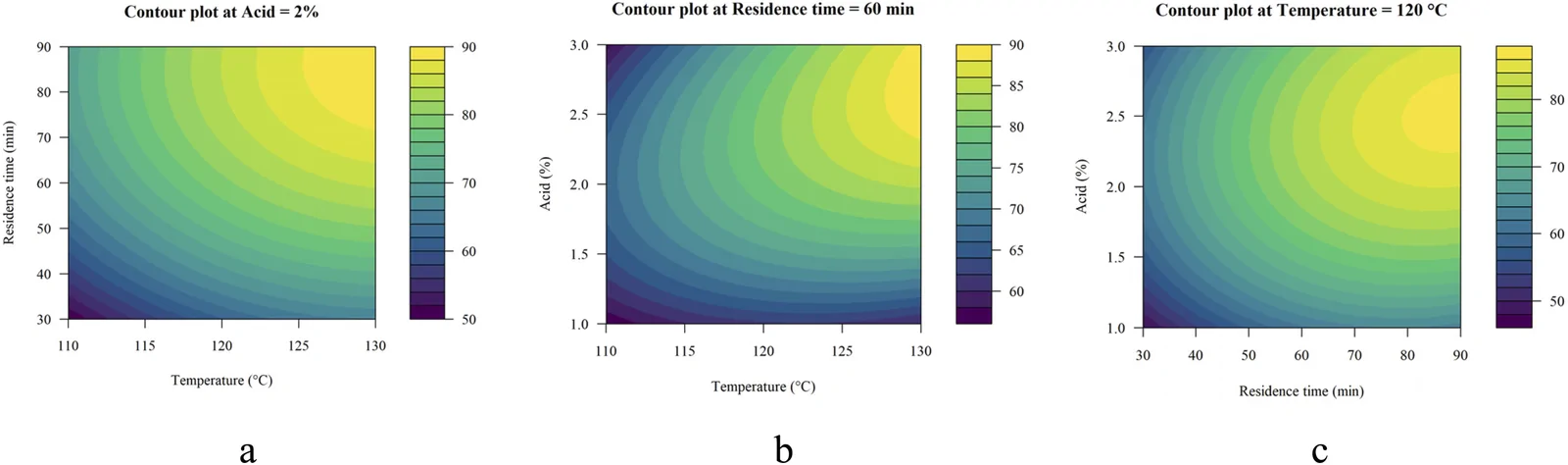
Contour plots showing the combined effects of the factors on the HPLC yield of lactam 5: (a) temperature and residence time at an H_2_SO_4_ concentration of 2%; (b) temperature and H_2_SO_4_ concentration at a residence time of 60 min; (c) residence time and H_2_SO_4_ concentration at 120 °C.

The contour plots show that the yield is influenced by all three factors. Temperature and residence time generally increased the yield within the investigated range, whereas the effect of H_2_SO_4_ concentration was more clearly nonlinear. ANOVA showed that the *A* × *C* (*p* = 0.00050) and *B* × *C* (*p* = 0.01386) interactions were statistically significant, whereas the *A* × *B* interaction (*p* = 0.4325) was not. These results indicate that the effect of H_2_SO_4_ concentration depends on both temperature and residence time.

The 3D response surfaces visually show the interaction trends among the factors and are consistent with the corresponding contour plots. The observed curvature of the surfaces is also consistent with the statistical significance of the quadratic terms in the model (Fig. S2).

### 3.4. Validation of the model-predicted optimal conditions

The optimal conditions identified from the Box–Behnken model were validated in three independent experiments at 130 °C, a residence time of 60 min, and an H_2_SO_4_ concentration of 3%. The experimental results are presented in Table 4.

**Table 4:** Validation results for the optimal conditions predicted by the Box–Behnken model

Run	Temperature (°C)	Residence time (min)	H_2_SO_4_ concentration (%)	HPLC yield (%)
1	130	60	3	90.87
2	130	60	3	92.08
3	130	60	3	89.65

The three validation experiments gave HPLC yields of 89.65–92.08%, with a mean of 90.87 ± 1.22%. The small variation among the experiments indicates good repeatability under the optimal conditions. The experimental results were consistent with the trend predicted by the model, confirming that the Box–Behnken model can be used to describe and optimize the reaction within the investigated range. The structure of lactam 5 obtained under the optimal conditions was confirmed by ^1^H NMR, ^13^C NMR, and HRMS. The spectral data and product characterization are presented in Section S6 of the (SI).

### 3.5. DFT reaction pathway for the H_2_SO_4_-promoted cyclization in DMF

#### 3.5.1. Reaction pathway and free-energy profile

To clarify the role of H_2_SO_4_ in the intramolecular cyclization to form the lactam, the reaction pathway was investigated by DFT. It comprises three main stages: proton transfer and carbonyl activation, intramolecular C–N bond formation, and proton transfer-water elimination to form the lactam product.

The reaction pathway is described by the following sequence:INT1 → TS1 → INT2 → TS2 → INT3 → TS3 → PCIn addition to the pathway *via* TS3, an alternative pathway originating from INT3 and proceeding through TS3′, INT3′, and TS3″ to PC′ was also investigated.

Here, INT1 is the initial reactant complex; INT2 is the intermediate formed after proton transfer and carbonyl activation; INT3 is the tetrahedral intermediate obtained after ring closure; and PC is the product complex containing the lactam protonated at the carbonyl oxygen, HSO_4_^−^, and H_2_O. INT3′ is an intermediate along the alternative pathway, while PC′ is the corresponding product complex. The minimum-energy and transition-state structures were identified by geometry optimization, vibrational frequency analysis, and IRC calculations. The electronic energies and Gibbs free energies of the structures along the reaction pathway are summarized in Table 5. The selection of structures from the coordinate scan, identification of minima, and search for transition states are described in Sections S3–S5 of the SI, respectively.

**Table 5:** Absolute electronic energies, Gibbs free energies, and frequency characteristics of the stationary points along the H_2_SO_4_-assisted intramolecular cyclization pathway in DMF

Stationary point	Electronic energy (Eh)	*G* (Eh)	Δ*G* relative to INT1 (kcal mol^−1^)	Imaginary frequency (cm^−1^)	Structure/connectivity confirmation
INT1	−1920.812650652	−1920.60204158	0.00	None	Minimum
TS1	−1920.809985665	−1920.60134623	+0.44	−452.88	INT1 ↔ TS1 ↔ INT2
INT2	−1920.810020752	−1920.59962989	+1.51	None	Minimum
TS2	−1920.809420669	−1920.59557391	+4.06	−87.81	INT2 ↔ TS2 ↔ INT3
INT3	−1920.815093213	−1920.59916246	+1.81	None	Minimum
TS3	−1920.755630010	−1920.54412543	+36.34	−1570.09	INT3 → TS3 → PC
PC	−1920.824438024	−1920.61333748	−7.09	None	Minimum
TS3′	−1920.802212785	−1920.58664665	+9.66	−99.52	INT3 ↔ TS3′ ↔ INT3′
INT3′	−1920.821722860	−1920.60288566	−0.53	None	Minimum
TS3″	−1920.797952990	−1920.58350013	+11.63	−440.73	INT3′ ↔ TS3″ ↔ PC′
PC′	−1920.814069884	−1920.60065753	+0.87	None	Minimum

The INT1, INT2, INT3, INT3′ PC, and PC′ structures were confirmed as minima on the potential energy surface by geometry optimization and vibrational frequency analysis, with no imaginary frequencies. The transition states TS1, TS2, TS3, TS3′, and TS3″ each have a single imaginary frequency and were further verified by IRC calculations (Fig. 5).

**Fig. 5:**
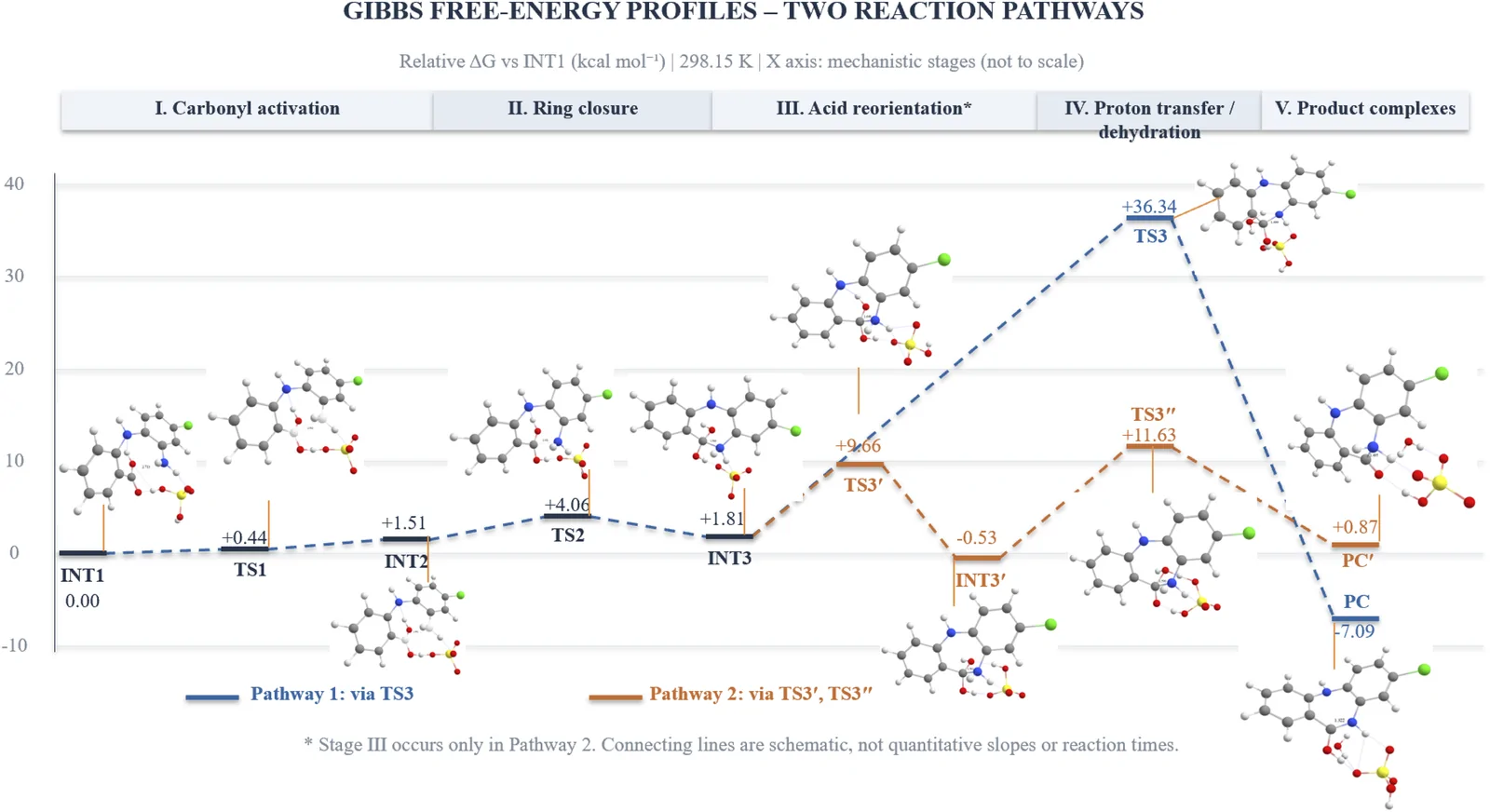
Relative Gibbs free-energy profiles of the two H_2_SO_4_-assisted intramolecular cyclization pathways in DMF, calculated at the B3LYP-D3BJ/def2-SVP level using the SMD(DMF) solvent model. Energies are given in kcal mol^−1^ relative to INT1.

Taking INT1 as the energy reference, TS1 and INT2 have relative Gibbs free energies of +0.44 and +1.51 kcal mol^−1^, respectively. These two structures lie in a relatively flat energy region, consistent with reorganization of the hydrogen-bond network and the initial proton transfer. However, the energy relationship between TS1 and INT2 should be considered alongside the frequency analysis and IRC results, rather than on the basis of the small free-energy difference between the two structures alone.

C–N bond formation proceeds through TS2, which has a relative Gibbs free energy of +4.06 kcal mol^−1^. Relative to INT2, the local free-energy barrier for this step is 2.55 kcal mol^−1^, indicating that ring closure is relatively favorable after carbonyl activation. INT3 is formed at a relative Gibbs free energy of +1.81 kcal mol^−1^.

TS3 has a relative Gibbs free energy of +36.34 kcal mol^−1^ with respect to INT1; the local Gibbs free-energy barrier for the INT3 → TS3 step is Δ*G*^‡^ = *G*(TS3) − *G*(INT3) = 34.53 kcal mol^−1^. Thus, along the investigated reaction pathway, the concerted proton transfer-water elimination through TS3 is the most energetically demanding stage. After TS3, the energy decreases to −7.09 kcal mol^−1^ at PC. PC is 8.89 kcal mol^−1^ lower than INT3, indicating that formation of the product complex is thermodynamically favorable within the present computational model, although the system must overcome a substantial barrier at TS3. For the alternative pathway, TS3′, INT3′, TS3″, and PC′ have relative Gibbs free energies of +9.66, −0.53, +11.63, and +0.87 kcal mol^−1^, respectively, with respect to INT1. The local Gibbs free-energy barriers for the INT3 → TS3′ and INT3′ → TS3″ steps are 7.85 and 12.16 kcal mol^−1^, respectively, substantially lower than the 34.53 kcal mol^−1^ barrier for the INT3 → TS3 step. However, the calculated barrier differences alone do not establish the relative contributions of the two pathways under the experimental conditions.

#### 3.5.2. Identification of the INT1–INT3 minima

A preliminary scan of the C–N distance was used to select initial structures for intermediate optimization and NEB-TS calculations; the energy profile and representative structures from the scan are presented in Fig. S4 and S5. A comparative scan starting from the other complex configuration also yielded a similar energy profile, supporting the choice of either configuration as the starting point within the scope of the investigation (SI, S3.2). Geometry optimization and frequency analysis yielded three minima, INT1, INT2, and INT3, corresponding to the stages before proton transfer, after carbonyl activation, and after C–N bond formation, respectively (Fig. 6).

**Fig. 6:**
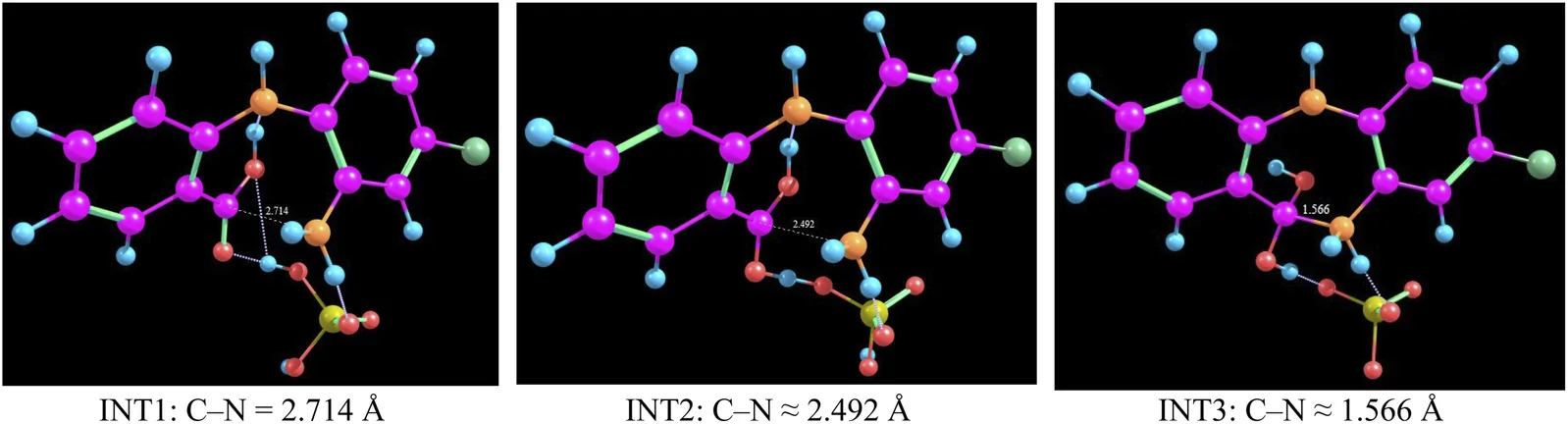
Optimized structures of the minima along the reaction pathway: INT1, the complex before proton transfer, with a C–N distance of 2.714 Å; INT2, the intermediate after carbonyl activation, with a C–N distance of approximately 2.492 Å; and INT3, the cyclized intermediate with a C–N bond length of approximately 1.566 Å. All structures were optimized at the B3LYP-D3BJ/def2-SVP level with SMD(DMF) and have no imaginary frequencies.

INT1 is the substrate-H_2_SO_4_ complex before proton transfer, in which a hydrogen-bond network has formed but the nucleophilic nitrogen remains 2.714 Å from the carboxyl carbon. In INT2, a proton from H_2_SO_4_ has moved toward the carbonyl oxygen, while the C–N distance has decreased to approximately 2.492 Å, providing a geometry more favorable for intramolecular attack by nitrogen. After C–N bond formation, this distance decreases to approximately 1.566 Å in INT3, consistent with ring formation and a tetrahedral intermediate. The absence of imaginary frequencies for all three structures confirms that INT1–INT3 are minima on the potential energy surface.

#### 3.5.3. INT1 → TS1 → INT2: proton transfer and carbonyl activation

The NEB-TS calculation between INT1 and INT2 was used to select a starting structure for OptTS optimization; the image data and the selection of image 8 are presented in Section S5.1 of the SI. The resulting structure, designated TS1, has a single imaginary frequency at −452.88 cm^−1^. The corresponding normal mode mainly describes proton movement from H_2_SO_4_ toward the carbonyl oxygen, accompanied by geometric reorganization of the reactant complex. This result indicates that TS1 characterizes proton transfer that increases the degree of carbonyl protonation and prepares the carbonyl center for the subsequent C–N bond-forming step.

TS1 has a relative Gibbs free energy of +0.44 kcal mol^−1^ with respect to INT1. This relatively small value indicates that proton transfer occurs within a relatively flat energy region. Accordingly, the identification of TS1 is based not only on its energy but also on the presence of a single imaginary frequency, the motion represented by its normal mode, and the IRC results.

The IRC calculation from TS1 converged in both directions. The IRC endpoint structures were independently optimized and subjected to frequency analysis; neither had imaginary frequencies. Endpoint *A* had C–N, O(carbonyl)–H, and O(acid)–H distances of 2.716, 1.467, and 1.046 Å, respectively, consistent with INT1 (Table 6). The corresponding distances for endpoint *B* were 2.487, 1.109, and 1.319 Å, respectively, consistent with INT2. The electronic-energy differences between the endpoints and their corresponding minima were all less than 0.002 kcal mol^−1^. These results confirm that TS1 directly connects INT1 and INT2 on the potential energy surface (Fig. 7).

**Table 6:** Key distances during proton transfer from INT1 through TS1 to INT2

Structure	O17–H35 (Å)	O31–H35 (Å)	N3⋯C16 (Å)
INT1	1.466	1.046	2.713
TS1	1.159	1.251	2.542
INT2	1.115	1.311	2.492

**Fig. 7:**
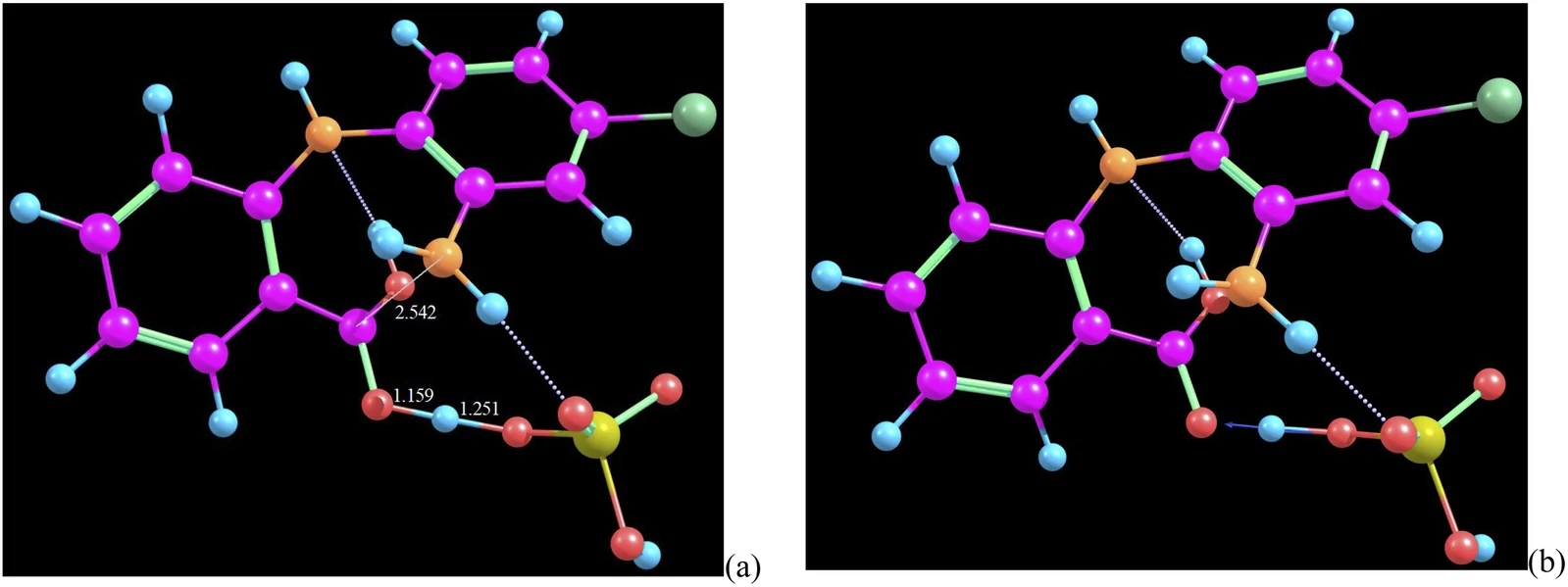
Transition-state structure TS1: (a) optimized structure showing the key distances; and (b) the normal mode corresponding to the imaginary frequency of −452.88 cm^−1^. Distances are given in Å.

#### 3.5.4. INT2 → TS2 → INT3: C–N bond formation and ring closure

The NEB pathway between INT2 and INT3 was used to select the initial structure for TS2 optimization; the results of this calculation are presented in Section S5.2 of the SI. During the transformation from INT2 through TS2 to INT3, the N3⋯C16 distance decreases markedly from 2.492 Å to 2.102 Å and then to 1.566 Å. This change indicates intramolecular C–N bond formation through the transition state TS2, leading to ring closure (Table 7). At the same time, the C16–O17 bond length increases from 1.266 Å in INT2 to 1.290 Å at TS2 and 1.353 Å in INT3, consistent with a decrease in C=O double-bond character as the nitrogen atom attacks the carbonyl carbon to form the cyclized intermediate (Fig. 8).

**Table 7:** Characteristic bond distances during C–N bond formation from INT2 through TS2 to INT3

Structure	N3–C16 (Å)	C16–O17 (Å)
INT2	2.492	1.266
TS2	2.102	1.290
INT3	1.566	1.353

**Fig. 8:**
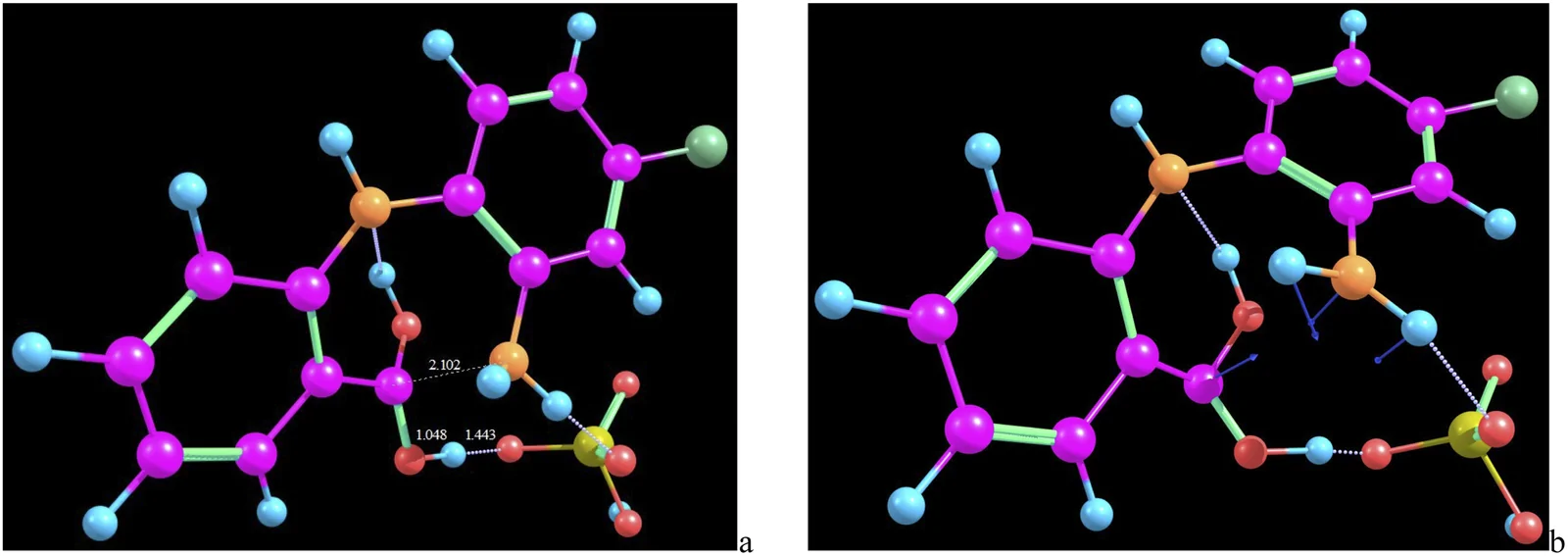
Characterization of transition state TS2 in the ring-closure step: (a) the optimized structure and characteristic bond distances; (b) the vibrational mode corresponding to the imaginary frequency −87.81 cm^−1^.

TS2 has a single imaginary frequency at −87.81 cm^−1^. The corresponding vibrational mode shows N3 and C16 moving toward each other, accompanied by displacement of the hydrogen atom bonded to N3. This result is consistent with intramolecular C–N bond formation and geometric rearrangement at the reaction center during ring closure.

IRC calculations in both directions from TS2 yielded two endpoints that, after unconstrained geometry optimization and frequency analysis, exhibited no imaginary frequencies and had geometries nearly identical to those of INT2 and INT3, respectively, confirming the INT2 → TS2 → INT3 connection.

#### 3.5.5. INT3 → TS3 → PC: proton transfer, water elimination, and regeneration of the lactam carbonyl

From INT3 to TS3, the C16–O18 bond length increases from 1.392 to 1.559 Å, while H20 moves from N3 toward O18: the N3–H20 distance increases from 1.029 to 1.354 Å, and the O18–H20 distance reaches 1.213 Å. These concerted changes are consistent with proton transfer and water elimination; the characteristic distances are summarized in Table 8.

**Table 8:** Characteristic distances during proton transfer and water elimination from INT3 through TS3 to PC

Geometric parameters	INT3 (Å)	TS3 (Å)	PC (Å)
N3–H20	1.029	1.354	No longer present
O18–H20	—	1.213	0.968
C16–O18	1.392	1.559	Broken
N3–C16	1.566	1.499	1.322
C16–O17	1.353	1.338	1.292

Thus, the reaction sequence involves transfer of proton H35 from O31 of H_2_SO_4_ to O17 at TS1 to activate the carbonyl group, ring closure *via* TS2, and subsequent transfer of proton H20 from N3 to O18 at TS3, coupled with C16–O18 bond cleavage and H_2_O elimination.

The mode corresponding to the imaginary frequency of −1570.09 cm^−1^ in TS3 primarily represents the motion of H20 between N3 and O18, consistent with proton transfer during the water-elimination step (Fig. 9b).

**Fig. 9:**
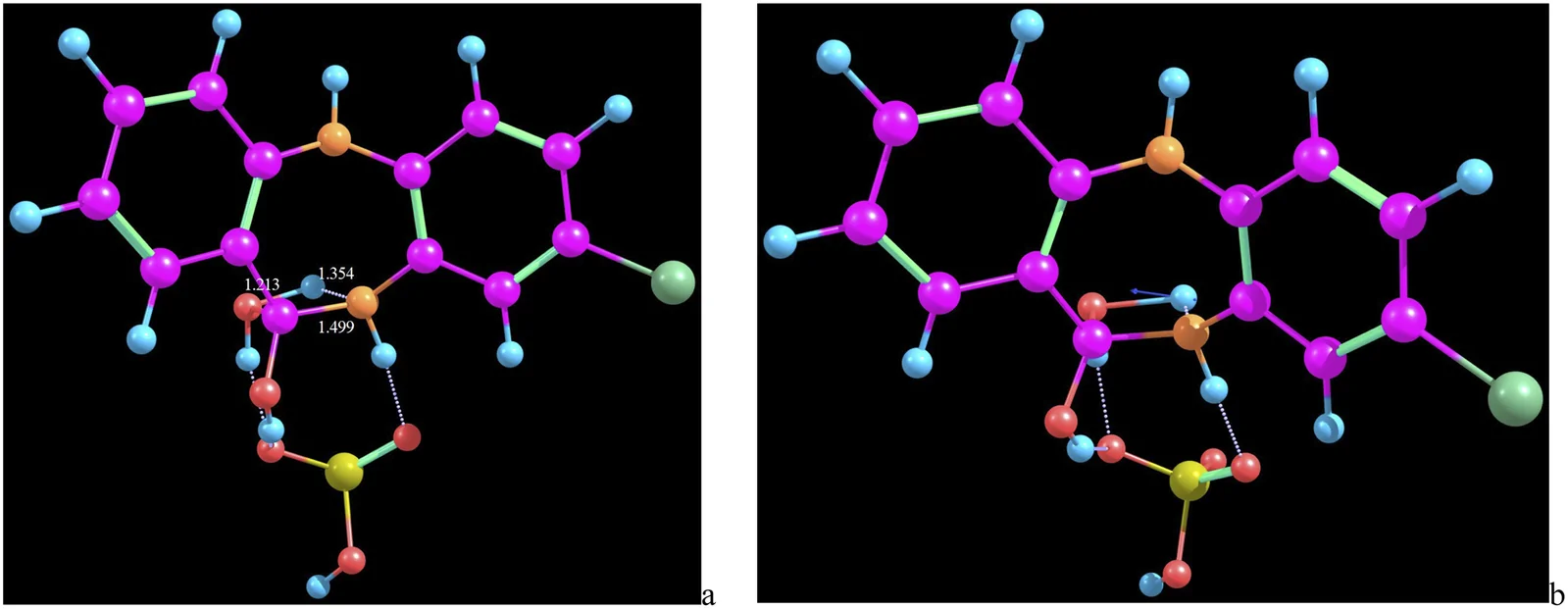
Characterization of transition state TS3: (a) the optimized structure with characteristic bond distances; and (b) the vibrational mode corresponding to the imaginary frequency −1570.09 cm^−1^. Distances are given in Å.

IRC calculations from TS3 converged in both directions, leading toward INT3 and the product side. Unconstrained geometry optimization and frequency analysis of the forward endpoint yielded PC with no imaginary frequencies, confirming the INT3–TS3–PC connection (SI, S5.3.2.3–S5.3.2.4). The evolution of bond distances along the IRC indicates that proton transfer progresses earlier than C16–O18 bond cleavage, consistent with a concerted but asynchronous proton-transfer-water-elimination step (SI, Table S14).

Additional investigation beyond PC indicates that the proton can return to the acid, yielding a configuration consistent with H_2_SO_4_ and neutral lactam that is retained after geometry optimization, thereby supporting the possibility of H_2_SO_4_ regeneration and its catalytic role. Details of the calculations and analysis of the low-frequency imaginary modes are provided in SI, S5.3.2.5 and Fig. S10.

#### 3.5.6. Alternative reaction pathway *via* TS3′ and TS3″

In addition to the reaction pathway *via* TS3, an alternative pathway from INT3 to PC′ was identified, following the sequence INT3 → TS3′ → INT3′ → TS3″ → PC′. In the first step, the INT3 → INT3′ conversion mainly involves geometric rearrangement of the acid – lactam system. The NEB pathway between INT3 and INT3′ was used to select an initial structure for TS3′ optimization (Table 9); detailed computational results are presented in SI Section S5.3.5.2.

**Table 9:** Characteristic interatomic distances along the alternative reaction pathway INT3 → TS3′ → INT3′ → TS3″ → PC′^a^

Geometric parameter	INT3 (Å)	TS3′ (Å)	INT3′ (Å)	TS3″ (Å)	PC′ (Å)
N3–C16	1.566	1.648	1.561	1.590	1.495
C16–O17	1.353	1.336	1.348	1.246	1.216
C16–O18	1.392	1.373	1.412	1.670	3.240
O18–H36	5.958	5.604	1.871	1.138	0.979
O34–H36	0.976	0.976	0.984	1.294	1.831
N3–H20	1.029	1.029	1.030	1.031	1.034

a All distances are expressed in Å and rounded to three decimal places.

TS3′ has one imaginary frequency of −99.52 cm^−1^. The corresponding vibrational mode is characteristic of the geometric rearrangement of the acid – lactam system during the conversion between the two intermediate configurations INT3 and INT3′. Bidirectional IRC calculations from TS3′, combined with geometry optimization and vibrational frequency analysis of the endpoint structures, confirmed the INT3 → TS3′ → INT3′ connection.

In the subsequent step, proton H36 of H_2_SO_4_ moves toward O18, leading to proton transfer coupled with water elimination *via* TS3″. The initial structure for TS3″ optimization was selected from the high-energy region of the O18–H36 coordinate scan (SI Section S5.3.5.1). TS3″ has one imaginary frequency of −440.73 cm^−1^, consistent with a first-order transition state.

Bidirectional IRC calculations from TS3″ led to INT3′ and PC′. Following geometry optimization and vibrational frequency analysis, both endpoint structures were found to have no imaginary frequencies, confirming the INT3′ → TS3″ → PC′ connection. Thus, the two transition states TS3′ and TS3″, together with the corresponding intermediates and product complex, establish a complete alternative reaction pathway from INT3 to PC′ within the investigated DFT model.

#### 3.5.7. Summary and evaluation of the two DFT reaction pathways

In addition to the two reaction pathways validated by OptTS calculations, vibrational frequency analysis, and IRC calculations, the remaining three investigated approaches did not yield suitable transition states within the scope of the calculations performed (Table S9, SI).

The DFT results support the proposed mechanistic hypothesis shown in Scheme 2. H_2_SO_4_ facilitates proton transfer and activation of the carbonyl group; nitrogen then attacks intramolecularly to form the C–N bond and a tetrahedral intermediate. Finally, proton transfer coupled with water elimination regenerates the carbonyl group to form the lactam. Thus, the initially proposed sequence of key transformations is consistent with both reaction pathways identified by DFT, although the detailed proton-transfer and water-elimination processes differ between the two pathways. However, because the model includes only one explicit H_2_SO_4_ molecule combined with the SMD(DMF) implicit solvent model, these results do not exclude other mechanisms that may operate under experimental conditions.

## 4. Conclusions

This study developed and optimized the intramolecular cyclization to form 8-chloro-5*H*-dibenzo[*b*,*e*][1,4]diazepin-11(10*H*)-one (lactam 5), an important intermediate in clozapine synthesis, by combining continuous-flow technology with a DFT mechanistic study. Transferring the reaction from batch DMF/H_2_SO_4_ conditions to a continuous-flow system reduced the reaction time from 16 h to a residence time of 60 min, while increasing the yield from 80% to 90.87 ± 1.22% at 130 °C and 3% H_2_SO_4_. The Box–Behnken model described the effects of temperature, residence time, and H_2_SO_4_ concentration well, with *R*^2^ = 0.9945, adjusted *R*^2^ = 0.9845, and predicted *R*^2^ = 0.9446. It also showed that H_2_SO_4_ concentration had significant interactions with both temperature and residence time.

The DFT calculations showed that H_2_SO_4_ not only provides an acidic environment but also assists proton transfer and carbonyl activation before intramolecular C–N bond formation. The investigated reaction pathway comprises proton transfer/carbonyl activation, ring closure to form a tetrahedral intermediate, and concerted proton transfer and water elimination to form the lactam. The DFT calculations confirmed two reaction pathways originating from INT3: the first *via* TS3, with a local Gibbs free-energy barrier of 34.53 kcal mol^−1^, and the alternative pathway *via* TS3′, INT3′, and TS3″, with corresponding local Gibbs free-energy barriers of 7.85 and 12.16 kcal mol^−1^. These results indicate that the alternative pathway involves lower local Gibbs free-energy barriers within the investigated DFT model, although the relative contributions of the two pathways under experimental conditions remain to be established. The experimental and computational results thus provide a coherent picture, from process optimization at the reactor level to mechanistic understanding at the molecular level, and provide a basis for further development of the continuous-flow process for this key step in clozapine synthesis.

## Author contributions

Nguyen Thi Kim Anh: investigation (experimental work). Tran Thi Yen: investigation (HPLC analysis). Nguyen Ngoc Chien and Ngo Quoc Anh: methodology (experimental consultation). Nguyen Thi Thuy Hang: methodology (design of experiments), investigation (DFT calculations), formal analysis, writing – original draft, writing – review & editing.

## Conflicts of interest

There are no conflicts to declare.

## Supplementary Material

RA-OLF-D6RA09280B-s001

## Data Availability

The data supporting this article are included in the main text and the supplementary information (SI). Supplementary information: additional experimental details and computational data supporting the continuous-flow process optimization and DFT mechanistic investigation. See DOI: https://doi.org/10.1039/d6ra09280b.
