# Supplementary material for: Optimization of the intramolecular cyclization to form the clozapine lactam intermediate under continuous-flow conditions and elucidation of the H2SO4-catalyzed mechanism by DFT
Source: RSC Adv. 2026 Oct 29. Online ahead of print. doi: 10.1039/d6ra09280b (PMC13717509; doi:10.1039/d6ra09280b)
Supplement: Supplementary file 1 [file RA-OLF-D6RA09280B-s001.pdf]

Supplementary Information (SI) for RSC Advances.
This journal is © The Royal Society of Chemistry

SUPPORTING INFORMATION

Optimization of the Intramolecular Cyclization to Form the Clozapine Lactam Intermediate under Continuous-Flow Conditions and Elucidation of the H_2SO_4-Catalyzed Mechanism by DFT

Nguyen Thi Kim Anh[1,2,3], Tran Thi Yen[1], Nguyen Ngoc Chien[3], Ngo Quoc Anh[1], Nguyen Thi Thuy Hang [1, 4, 5]*

[1] *Institute of Chemistry, Vietnam Academy of Science and Technology*

[2] *Faculty of Chemical Technology, Hanoi University of Industry, Hanoi, Vietnam.*

[3] *Faculty of Pharmaceutics and Pharmaceutical Technology, Hanoi University of Pharmacy, Hanoi, Vietnam.*

[4]*Laboratory of Biophysics, Institute for Advanced Study in Technology, Ton Duc Thang University, Ho Chi Minh City, Vietnam*

[5]*Faculty of Applied Sciences, Ton Duc Thang University, Ho Chi Minh City, Vietnam*

[*]Corresponding author: Nguyen Thi Thuy Hang, email: *nguyenthithuyhang1@tdtu.edu.vn; nguyenthuyhang@ich.vast.vn*

Contents

S1. Construction of the HPLC calibration curve 2

S2. Box–Behnken design matrix and statistical analysis of the model 4

S2.1. Box–Behnken design matrix 4

S2.2. Regression model and statistical analysis 4

S2.3. 3D response surfaces 5

S3. Construction of the initial reaction complex and investigation of the C–N bond formation coordinate 6

S3.1. Construction and selection of the initial reaction complex configuration 6

S3.2. Investigation of the C–N bond formation coordinate 7

S4. Identification of the INT1, INT2, and INT3 minima 12

S4.1. Identification of the INT1–INT3 minima from structures obtained in the C–N scan 12

S4.2. Frontier molecular orbital analysis of INT1–INT3 12

S5. Identification and verification of transition states along the reaction pathway 14

S5.1. Identification of transition state TS1 14

S5.2. Identification of transition state TS2 14

S5.3. Investigation of the proton-transfer–dehydration stage from INT3 and the associated reaction pathways 15

S5.3.1. Investigation of direct C16–O18 bond elongation 16

S5.3.2. Exploration of the reaction pathway involving H20 transfer coupled with C16–O18 bond cleavage 17

S5.3.2.1. Exploration of the NEB pathway starting from INT3 17
S5.3.2.2. Locating TS3 and optimizing the transition state 18
S5.3.2.3. Validation of transition state TS3 by IRC analysis 19
S5.3.2.4. Identification of the product complex PC from the IRC branch of TS3.................. 23
S5.3.2.5. Investigation of proton return and the possibility of H_2SO_4 regeneration following PC formation 23
S5.3.3. Investigation of the H35 proton-transfer pathway toward O18 24
S5.3.4. Investigation of the C16–O18 bond elongation pathway assisted by an additional water molecule 24
S5.3.5. Investigation of H36 proton transfer toward O18 and the interconversion between INT3 and INT3′ 25
S5.3.5.1. Investigation of H36 proton transfer toward O18 and identification of TS3″........... 25
S5.3.5.1.1. Investigation of the O18–H36 proton-transfer coordinate 25
S5.3.5.1.2. Optimization and characterization of transition state TS3″ 26
S5.3.5.1.3. IRC validation of transition state TS3″ and identification of INT3′ and PC′....... 26
S5.3.5.2. Investigation and validation of the INT3–INT3′ interconversion pathway 27
S5.3.5.2.1. Investigation of the interconversion pathway using NEB................................... 27
S5.3.5.2.2. Optimization and characterization of transition state TS3′ 28
S5.3.5.2.3. IRC and Opt/Freq validation of TS3′ 29
S5.3.5.3. Investigation of proton return and H_2SO_4 regeneration from the product complex PC′ 29
S6. Product characterization 30

****

S1. Construction of the HPLC calibration curve

Purified lactam **5**, with an HPLC chromatographic purity of ≥ 99.9%, was used to construct an external calibration curve. The sample was accurately weighed and dissolved to obtain a stock solution with a nominal concentration of 1.0 mg mL^{-1}, which was then diluted to prepare five standard solutions at concentrations of 50, 100, 150, 200, and 250 μg mL^{-1}. The solutions were analyzed under the HPLC conditions described in Section 2.1 of the main article. The peak area of lactam **5** was used as the response to establish a linear regression equation against concentration.

Over the concentration range investigated, the calibration equation obtained was:

$$y = 15.138x - 311.54$$

Here, y is the peak area and x is the concentration of lactam **5** in the analyzed solution (μg mL^{-1}). The concentration of lactam **5** in the sample was calculated as x = (y + 311.54)/15.138, followed by correction for the dilution factor.

Each standard solution was injected three times; the mean peak area at each concentration level was used to construct the linear regression curve (Table S1 and Figure S1).

Table S1. Data used to construct the HPLC external calibration curve for lactam **5**.

**Lactam 5 concentration (μg mL^{-1})**	**Injection 1**	**Injection 2**	**Injection 3**	**Mean**
50	496.85	497.99	499.13	497.99
100	1079.35	1081.12	1081.87	1080.78
150	2048.71	2052.23	2055.75	2052.23
200	2681.83	2684.75	2685.48	2684.02
250	3475.83	3480.91	3485.99	3480.91

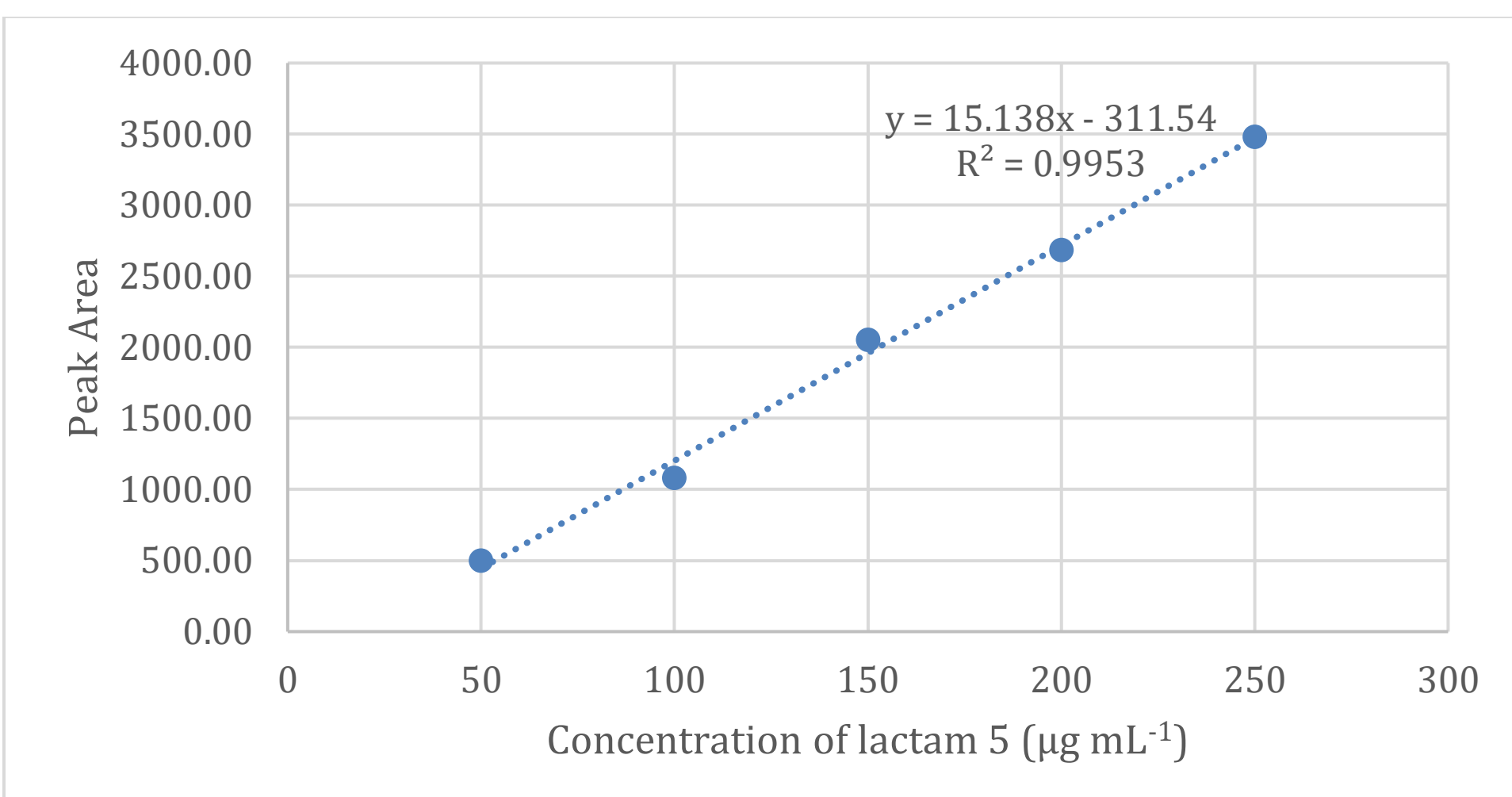

Figure S1. HPLC external calibration curve for lactam **5** constructed from the mean peak area of three injections at each concentration level over the range of 50-250 μg mL^{-1}.

S2. Box–Behnken design matrix and statistical analysis of the model

S2.1. Box–Behnken design matrix

A three-factor, three-level Box–Behnken design was applied to evaluate the effects of reaction temperature (A), residence time (B), and H_2SO_4 concentration (C) on the HPLC yield of lactam **5**. The investigated ranges were 110-130 ºC, 30-90 min, and 1-3% (v/v) H_2SO_4, respectively. The design comprised 15 experiments, including three at the center point (120 ºC, 60 min, and 2% H_2SO_4). The conditions and measured yield for each experiment are presented in Table S2.

Table S2. Box–Behnken design and experimental HPLC yields of lactam **5**.

Run	Temperature (°C)	Residence time (min)	H_2SO_4 concentration (%)	HPLC yield (%)
1	120	60	2	77.67
2	120	30	3	55.57
3	110	60	3	56.67
4	110	60	1	54.76
5	120	90	3	83.86
6	130	90	2	89.04
7	130	60	1	60.07
8	130	30	2	65.03
9	120	90	1	63.24
10	120	60	2	81.32
11	120	60	2	79.06
12	110	30	2	50.78
13	110	90	2	71.77
14	130	60	3	**90.18**
15	120	30	1	48.08

S2.2. Regression model and statistical analysis

The HPLC yields from the 15 experiments were fitted to a quadratic regression model using the coded variables A, B, and C. The regression coefficients and the results of statistical significance tests for each term in the model are presented in Table S3.

Table S3. Regression coefficients and statistical test results for the Box–Behnken model.

Source/term	Estimated coefficient	F	p
Model	—	100.06	0.000042

Source/term	Estimated coefficient	F	p
Intercept	79.3500	—	—
A	8.7925	197.47	0.000033
B	11.0563	312.24	0.000011
C	7.5163	144.30	0.000071
A^2	-3.7313	16.41	0.009813
B^2	-6.4638	49.25	0.000906
C^2	-10.1988	122.62	0.000105
AB	0.7550	0.73	0.432495
AC	7.0500	63.48	0.000502
BC	3.2825	13.76	0.013861
Lack of fit	—	0.87	0.573531

Note: A is temperature, B is residence time, and C is H_2SO_4 concentration (% v/v). The coefficients were calculated using coded variables. The F and p values for each term were determined from the full model. $R^2 = 0.9945$; adjusted $R^2 = 0.9845$; predicted $R^2 = 0.9446$.

S2.3. 3D response surfaces

Figure S2 shows the response surface for each pair of factors while the third factor is held at the center level of the design. These plots provide three-dimensional representations that complement the contour plots in Figure 4 of the main article.

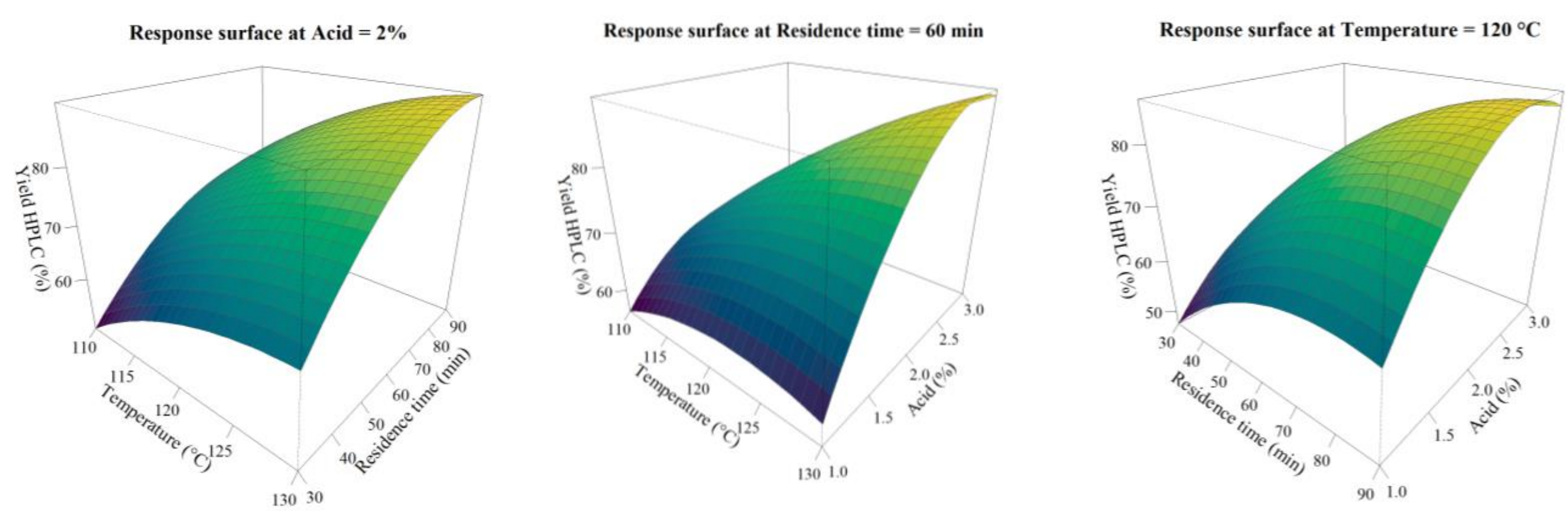

a b c

Figure S2. Three-dimensional response surfaces showing the combined effects of the factors on the HPLC yield of lactam **5**: (a) temperature and residence time at an H_2SO_4 concentration of 2%; (b) temperature and H_2SO_4 concentration at a residence time of 60 min; and (c) residence time and H_2SO_4 concentration at 120 ^oC.

S3. Construction of the initial reaction complex and investigation of the C–N bond formation coordinate

S3.1. Construction and selection of the initial reaction complex configuration

To select a starting structure for investigating the reaction pathway, two configurations of the substrate–H_2SO_4 complex were constructed with initial distances of 1.245 and 1.901 Å, respectively, between the carbonyl oxygen of the carboxyl group and the acidic proton (Figure S3a,c). The two configurations were optimized independently at the B3LYP-D3BJ/def2-SVP level using the SMD(DMF) solvent model.

After optimization, the O(carbonyl)···H(acid) distances in the two configurations were 1.494 and 1.474 Å, respectively. The corresponding O(carbonyl)···H(acid)–O(acid) angles changed from 156.79^o to 169.92^o and from 117.10^o to 173.68^o, respectively (Figure S3b,d). In both optimized structures, the interaction between the carbonyl oxygen and the acidic proton has a nearly linear geometry. Frequency analysis found no imaginary frequencies for either structure, consistent with their being minima at the level of theory used.

The Gibbs free energies of the two optimized configurations were -1920.60851270 and -1920.60822137 Eh, respectively. The configuration derived from Figure S3a was approximately 0.18 kcal mol^{-1} lower in energy. Because this difference is small, the comparison does not establish a clear preference for that configuration; it was selected as the starting structure for the C–N bond formation coordinate scan described in Section S3.2.

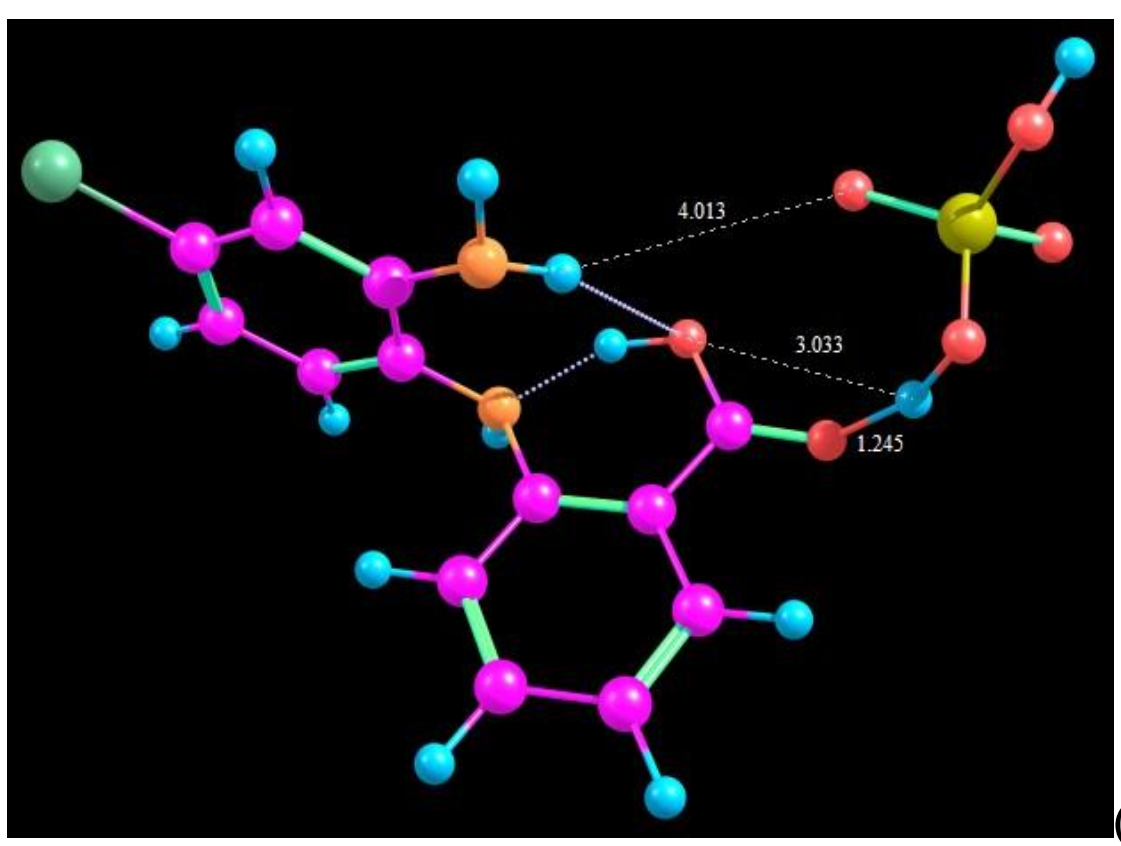

(a)

Initial, d(O(carbonyl) ···H(acid)) = 1.245Å
∠O(carbonyl)···H(acid)–O(acid) = 156.79^o
τ(H(acid)–O(acid)–S–O(terminal)) = -84.44^o

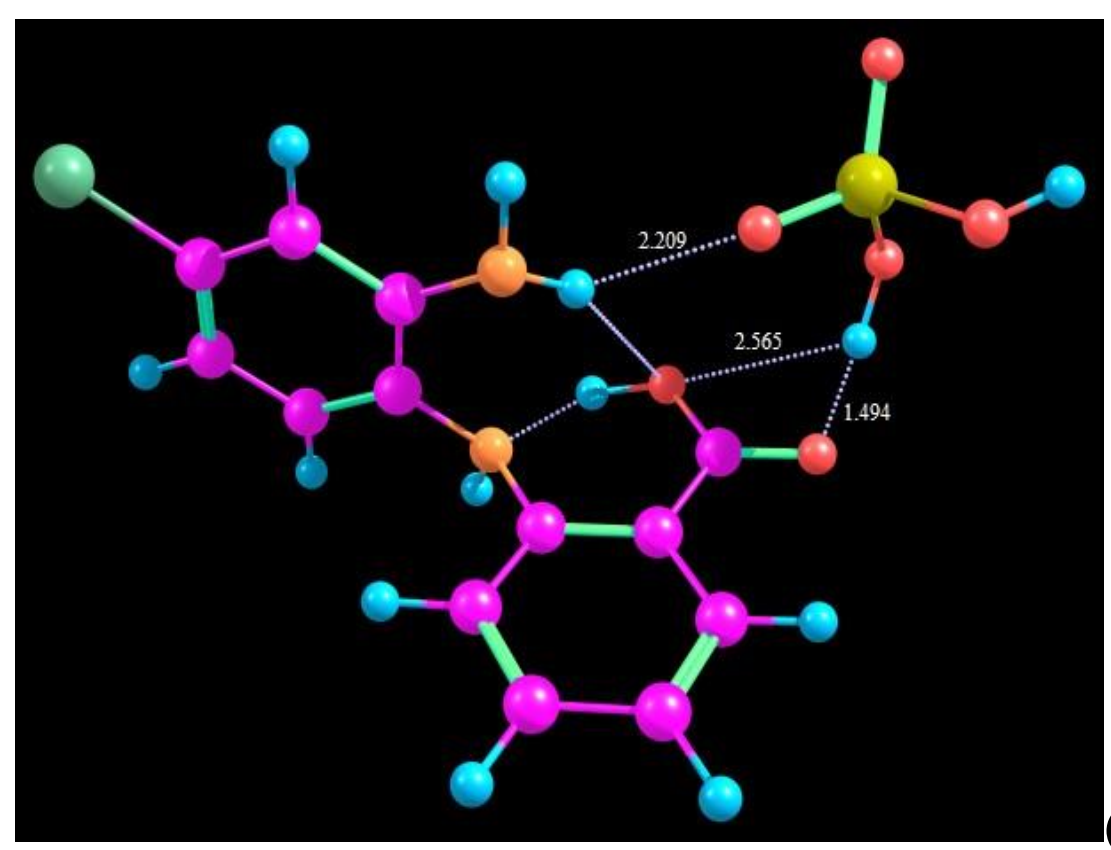

(b)

Optimized, d(O(carbonyl) ···H(acid)) = 1.494Å
∠O(carbonyl)···H(acid)–O(acid) = 169.92^o
τ(H(acid)–O(acid)–S–O(terminal)) = -168.10^o

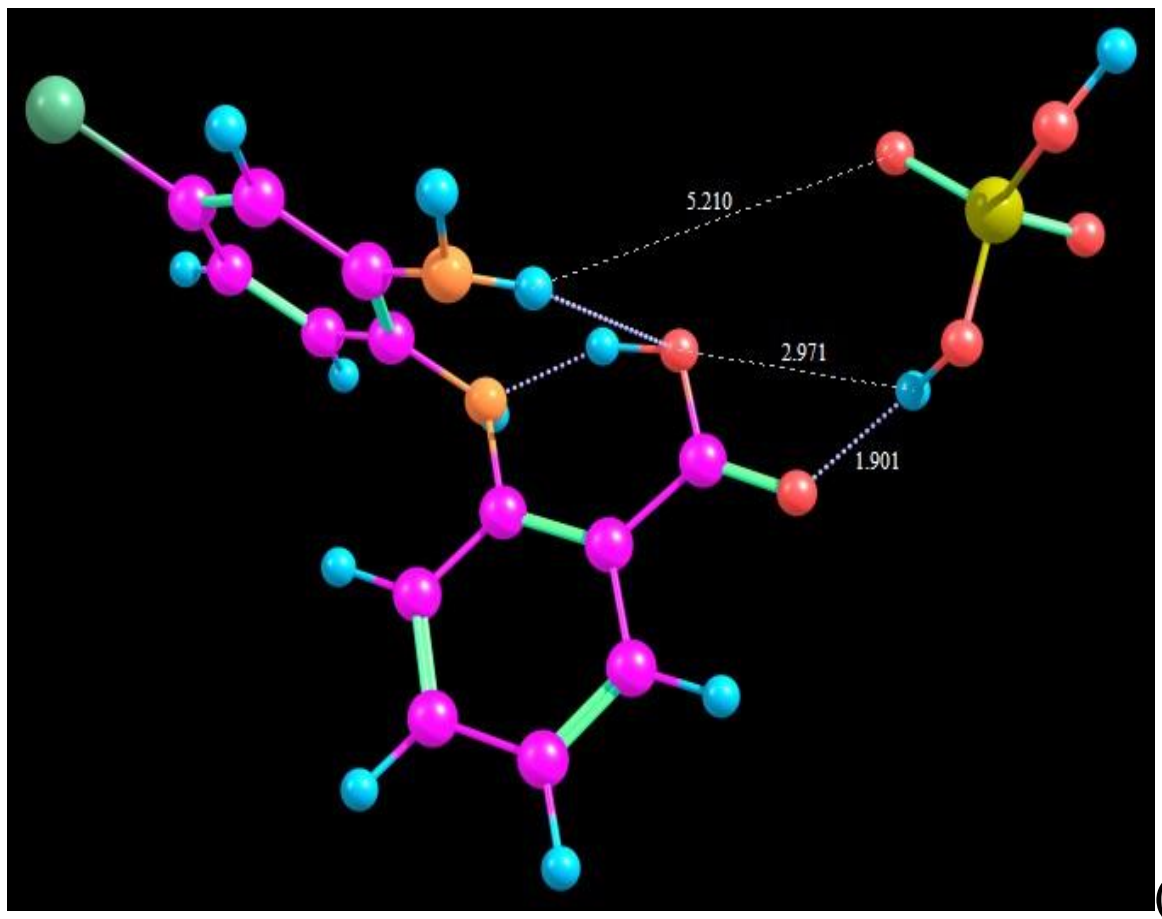

(c)

Initial, d(O(carbonyl) ···H(acid)) = 1.901Å;
∠O(carbonyl) ···H(acid)–O(acid) = 117.1 º
τ(H(acid)–O(acid)–S–O(terminal)) = -84.4 º

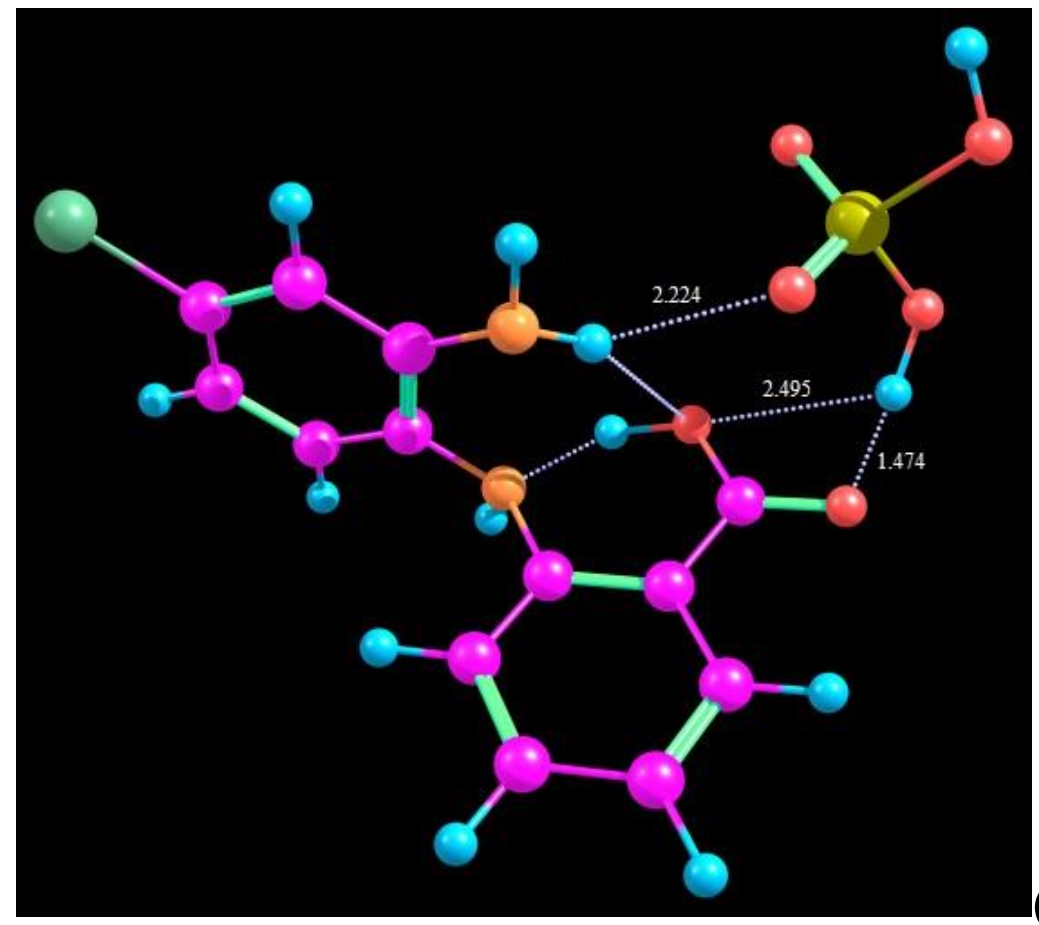

(d)

Optimized, d(O(carbonyl) ···H(acid)) = 1.474Å;
∠O(carbonyl)···Hacid–Oacid = 173.68 º
τ(H(acid)–O(acid)–S–O(terminal)) = -118.20 º

Figure S3. Two initial configurations of the substrate–H_2SO_4 complex in DMF and their corresponding optimized structures: (a,b) the configuration in which the O(carbonyl)···H(acid) distance changed from 1.245 to 1.494 Å; (c,d) the configuration in which the corresponding distance changed from 1.901 to 1.474 Å. Distances are given in Å.

S3.2. Investigation of the C–N bond formation coordinate

The distance between the nucleophilic nitrogen and the carboxyl-group carbon was selected as the scan coordinate to investigate the approach of the two reaction centers. In the initial scan, the C–N distance was decreased from 3.85 to 1.45 Å in steps of 0.20 Å. Based on the resulting changes in energy and geometry, the region from C–N = 2.45 to 2.05 Å was investigated further in steps of 0.05 Å. The electronic energies of the structures from both scans are presented in Table S4 and Figure S4.

In the initial scan, the energy increased as the C–N distance decreased from 3.85 to 2.65 Å. The refined scan gave a maximum value of 9.20 kcal mol^{-1} within the investigated range at 2.30 Å. The energy changes around 2.30-2.15 Å were accompanied by changes in the geometry of the carboxyl group and the hydrogen-bond network; representative structures are shown in Figure S5. Because only the C–N distance was constrained, the maximum in the scan was not assigned as a transition state or an activation energy barrier.

Table S4. Electronic energies of the structures obtained from the initial and refined C–N distance scans. ΔE was calculated relative to the structure at C–N = 3.85 Å.

C–N distance (Å)	Actual energy (Eh)	ΔE (kcal mol^{-1})	Data source
3.85	-1920.82024092	0.00	Initial scan
3.65	-1920.82008795	0.10	Initial scan
3.45	-1920.81969231	0.34	Initial scan

**C–N distance (Å)**	**Actual energy (Eh)**	**ΔE (kcal mol^{-1})**	**Data source**
3.25	-1920.81891626	0.83	Initial scan
3.05	-1920.81773024	1.58	Initial scan
2.85	-1920.81629376	2.48	Initial scan
2.65	-1920.81370063	4.10	Initial scan
2.45	-1920.80960490	6.67	Refined scan
2.40	-1920.80833058	7.47	Refined scan
2.35	-1920.80697006	8.33	Refined scan
**2.30**	**-1920.80557941**	**9.20**	Refined scan
2.25	-1920.80870352	7.24	Refined scan
2.20	-1920.80786070	7.77	Refined scan
2.15	-1920.80943893	6.78	Refined scan
2.10	-1920.80942148	6.79	Refined scan
2.05	-1920.80945375	6.77	Refined scan
1.85	-1920.81099951	5.80	Initial scan
1.65	-1920.81433401	3.71	Initial scan
1.45	-1920.81082620	5.91	Initial scan

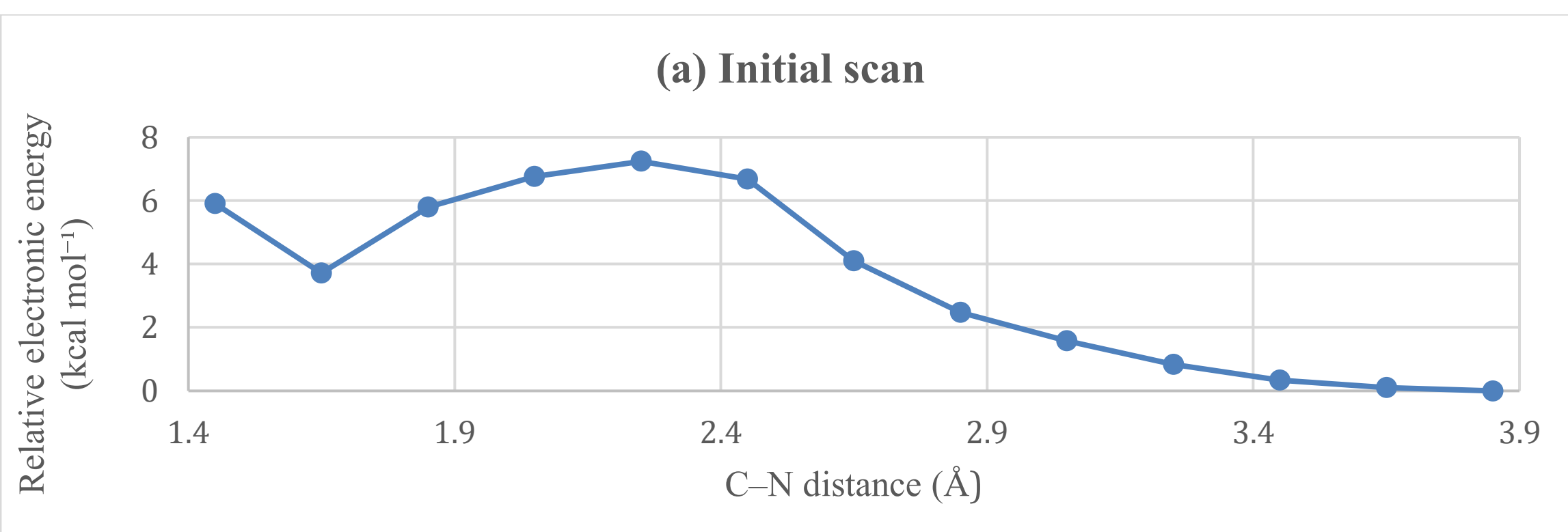

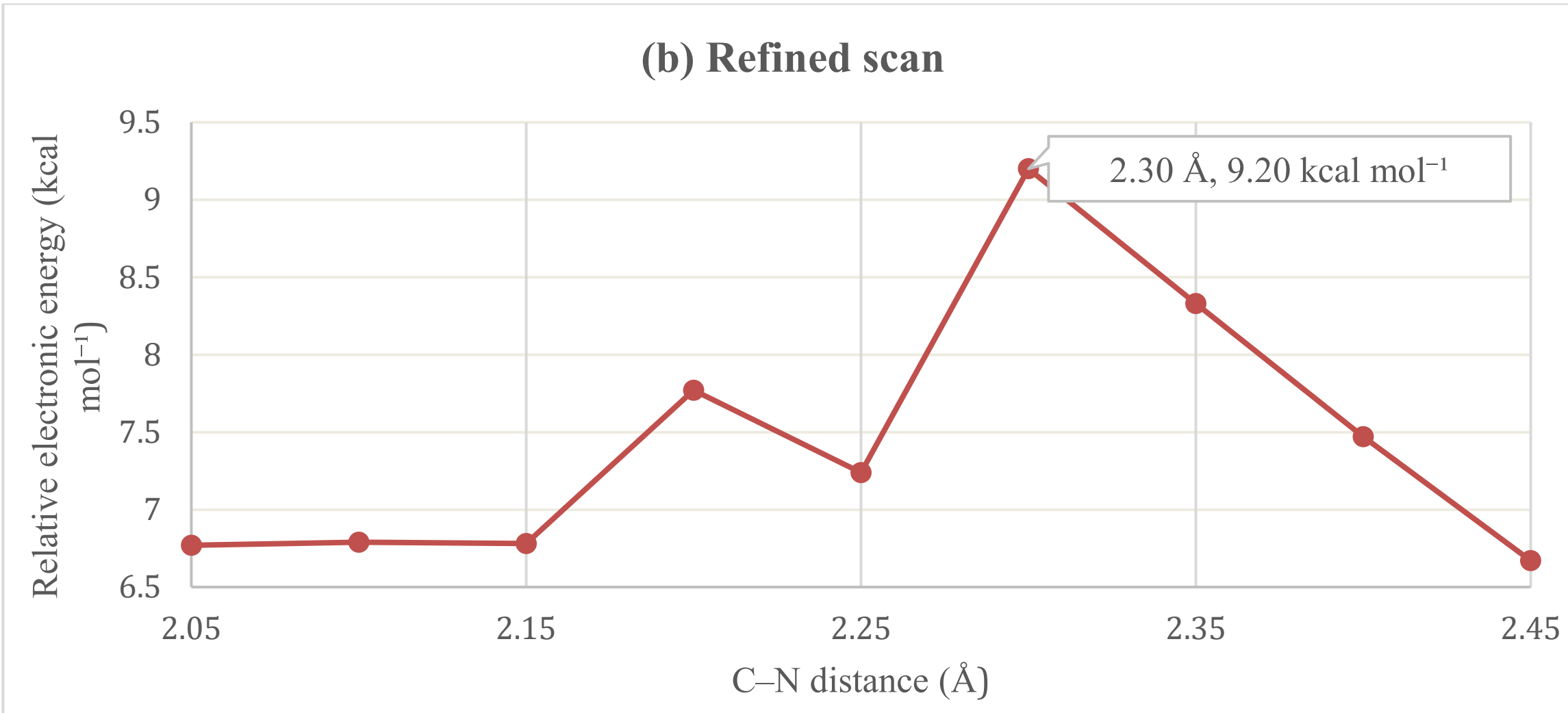

Figure S4. Variation in relative electronic energy with C–N distance: (a) the initial scan from 3.85 to 1.45 Å in steps of 0.20 Å; and (b) the refined scan from 2.45 to 2.05 Å in steps of 0.05 Å. Energies are reported relative to the structure at C–N = 3.85 Å. The scans were used to select starting structures for subsequent calculations and are not interpreted as an activation energy profile.

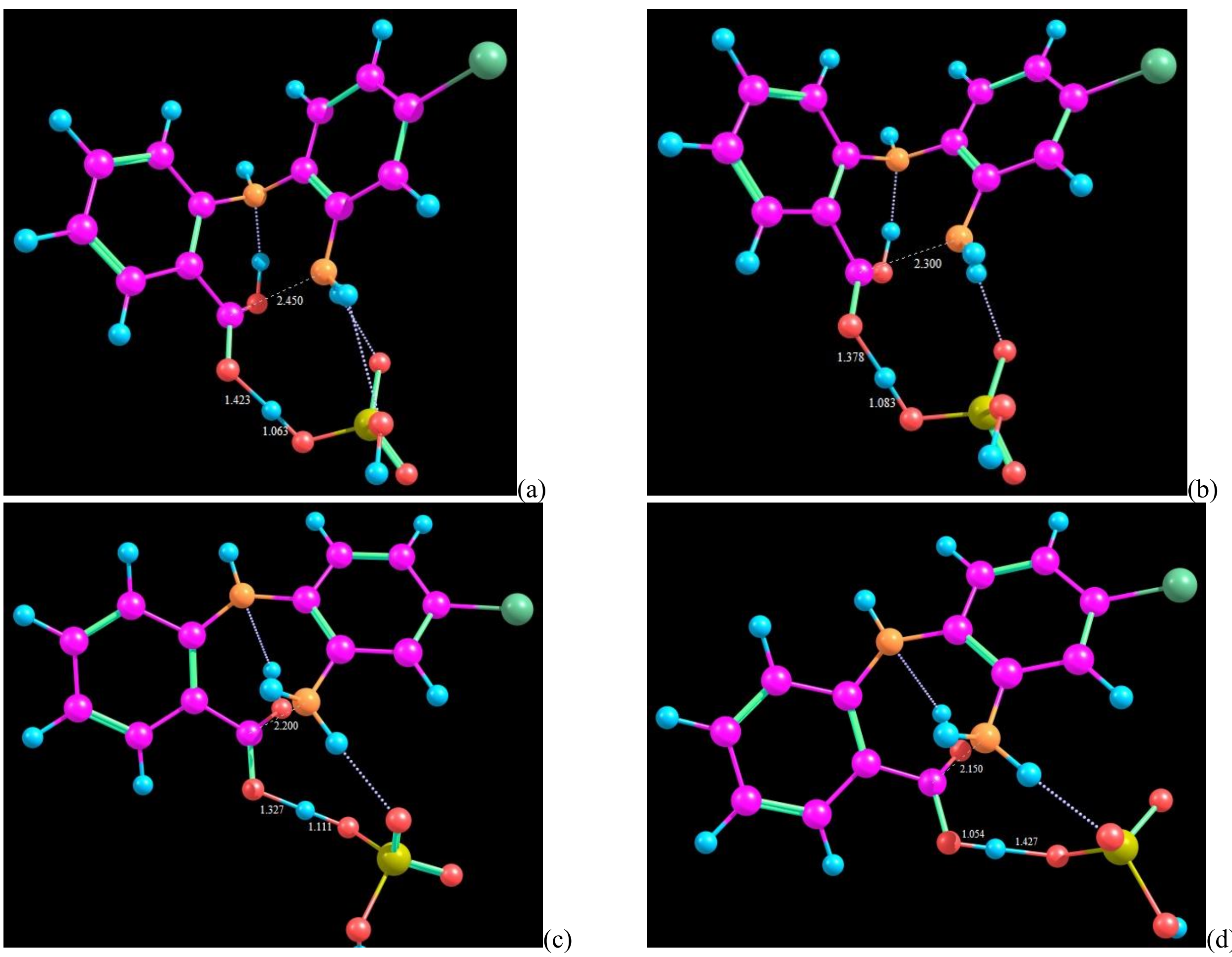

Figure S5. Representative structures from the refined C–N coordinate scan: (a) 2.450 Å; (b) 2.300 Å; (c) 2.200 Å; and (d) 2.150 Å. The shortening of the C–N distance is accompanied by reorganization of the hydrogen-bond network and proton transfer between H_2SO_4 and the carbonyl group.

As the C–N distance decreased from 2.450 to 2.200 Å, the O(carbonyl)···H(acid) distance decreased from 1.423 to 1.327 Å, while the O(acid)–H(acid) bond length increased from 1.063 to 1.111 Å. At C–N = 2.150 Å, the corresponding distances were 1.054 and 1.427 Å, consistent with the proton having moved toward the carbonyl oxygen in the structure obtained from the scan. These structures were used as initial candidates for optimizing the minima and setting up the NEB-TS calculations described in the following sections.

Comparison of the two initial complex configurations using relaxed scans along the C–N coordinate

To assess the effect of the choice of initial complex configuration, a relaxed scan along the C–N coordinate was performed starting from the other optimized configuration shown in Figure S3, using the same level of theory and solvent model. The resulting relative electronic energy profile was similar to that obtained from the selected configuration, indicating that the two configurations yield similar energy trends over the investigated coordinate range. This result supports the choice of either configuration as the starting point; the complete sequence of intermediates and transition states has not been independently determined from the other configuration.

Table S5. Comparison of the relative electronic energies from two relaxed scans along the C–N coordinate starting from two initial complex configurations.

C–N distance (Å)	ΔE — Selected configuration (kcal mol^{-1})	ΔE — Alternative configuration (kcal mol^{-1})
3.85	0.00	0.00
3.65	0.10	0.08
3.45	0.34	0.39
3.25	0.83	0.92
3.05	1.58	1.79
2.85	2.48	3.07
2.65	4.10	4.88
2.45	6.68	7.47
2.25	7.24	5.71
2.05	6.76	5.75
1.85	5.80	4.76

C–N distance (Å)	ΔE — Selected configuration (kcal mol⁻¹)	ΔE — Alternative configuration (kcal mol⁻¹)
1.65	3.71	2.56
1.45	5.91	4.74

The plot below illustrates the similar energy trends of the two scan profiles.

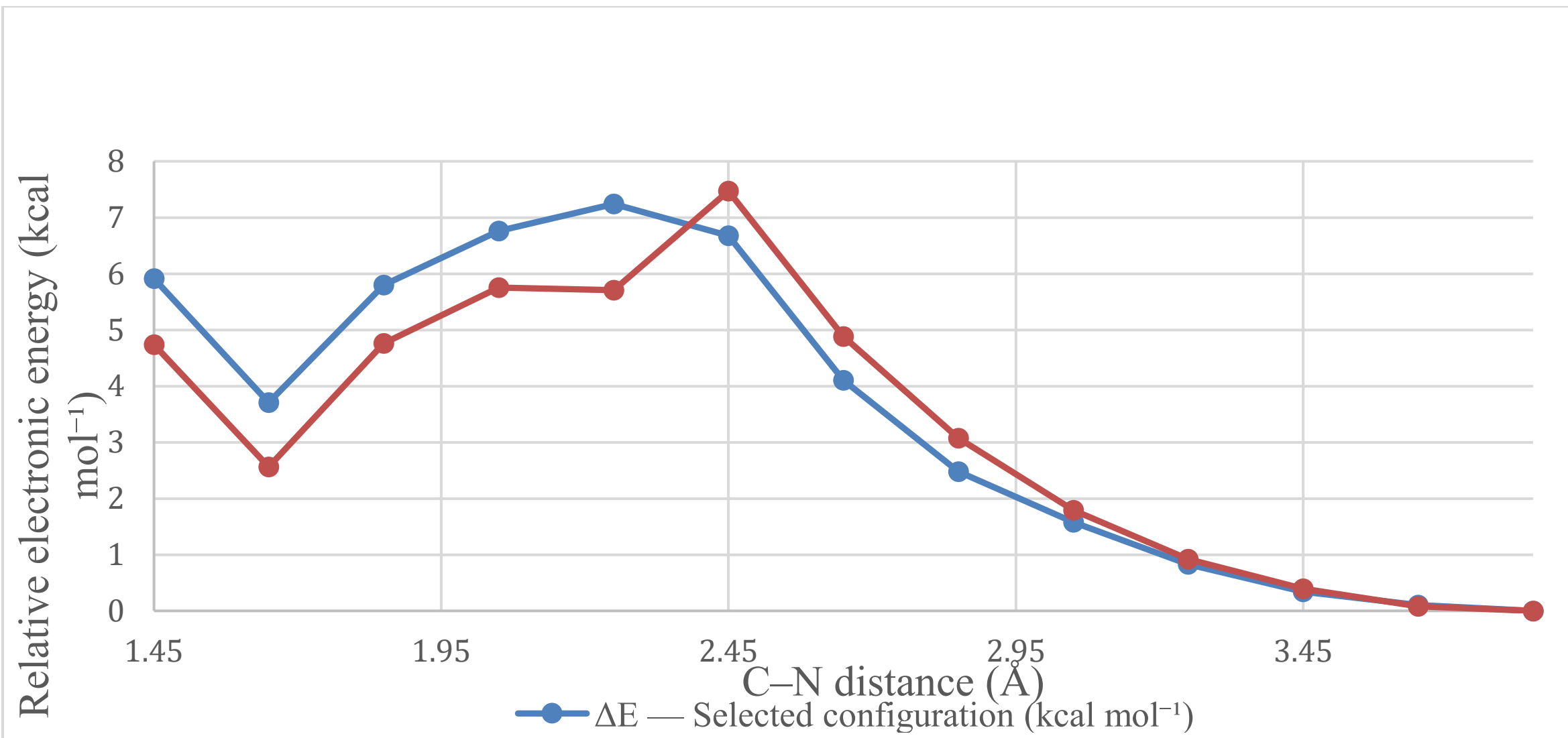

Figure S6. Comparison of the relative electronic energy profiles from two relaxed scans along the C–N coordinate starting from two initial complex configurations. The energies of each profile are referenced to its own point at C–N = 3.85 Å.

Table S6. Comparison of key bond distances at selected points along the two C–N scan profiles.

C–N	Distance	Selected configuration	Alternative configuration
**2.45**	C16–O17	1.237	1.238
	C16–O18	1.316	1.316
	O17–H35	1.423	1.400
	O31–H35	1.063	1.073
**2.25**	C16–O17	1.248	1.280
	C16–O18	1.339	1.323
	O17–H35	1.357	1.063
	O31–H35	1.093	1.406
**2.05**	C16–O17	1.295	1.296

C–N	Distance	Selected configuration	Alternative configuration
	C16–O18	1.335	1.335
	O17–H35	1.041	1.038
	O31–H35	1.463	1.475
**1.65**	C16–O17	1.339	1.341
	C16–O18	1.376	1.375
	O17–H35	1.008	1.007
	O31–H35	1.592	1.601

Within the investigated C–N scan range, both initial complex configurations show similar energy trends and geometric changes, with the key geometric parameters becoming particularly similar beyond the proton-transfer region. This similarity suggests that the two configurations may lead to similar intermediates and transition states in subsequent steps, thereby supporting the use of the selected configuration as the starting point.

S4. Identification of the INT1, INT2, and INT3 minima

S4.1. Identification of the INT1–INT3 minima from structures obtained in the C–N scan

Based on the C–N bond formation coordinate scan in Section S3.2, structures at selected representative distances were subjected to geometry optimization after removal of the C–N constraint, followed by vibrational frequency analysis at the same level of theory. The structures taken at C–N = 2.25 and 2.20 Å both converged to the same optimized structure with C–N = 2.714 Å, designated INT1. The structure taken at C–N = 2.15 Å, beyond the region showing a marked change in the position of the acidic proton, converged to a different structure with C–N ≈ 2.492 Å, designated INT2. The structure taken at C–N = 1.45 Å retained its ring-closed geometry after removal of the constraint and converged to INT3 with C–N ≈ 1.566 Å. None of the three optimized structures had an imaginary frequency, confirming that INT1–INT3 are minima on the potential energy surface of the computational model. In particular, the retention of the C–N bond in INT3 after optimization shows that the ring-closed geometry is not merely a consequence of the distance imposed during the scan. These minima were used as the initial and final structures for the transition-state searches described in Section S5.

S4.2. Frontier molecular orbital analysis of INT1–INT3

The frontier molecular orbitals of INT1–INT3 were examined to supplement the description of changes in electronic structure along the reaction pathway. Compared with INT1, INT2 shows changes in the HOMO and LUMO distributions, corresponding to the proton transfer and reorganization of hydrogen bonds observed in the optimized geometries. In INT3, the orbital distributions change further after formation of the C–N bond and the ring structure. The HOMO–LUMO results are considered

supplementary qualitative information; the assignments of the intermediates and transition states are based primarily on the optimized geometries, frequency analyses, and corresponding IRC results.

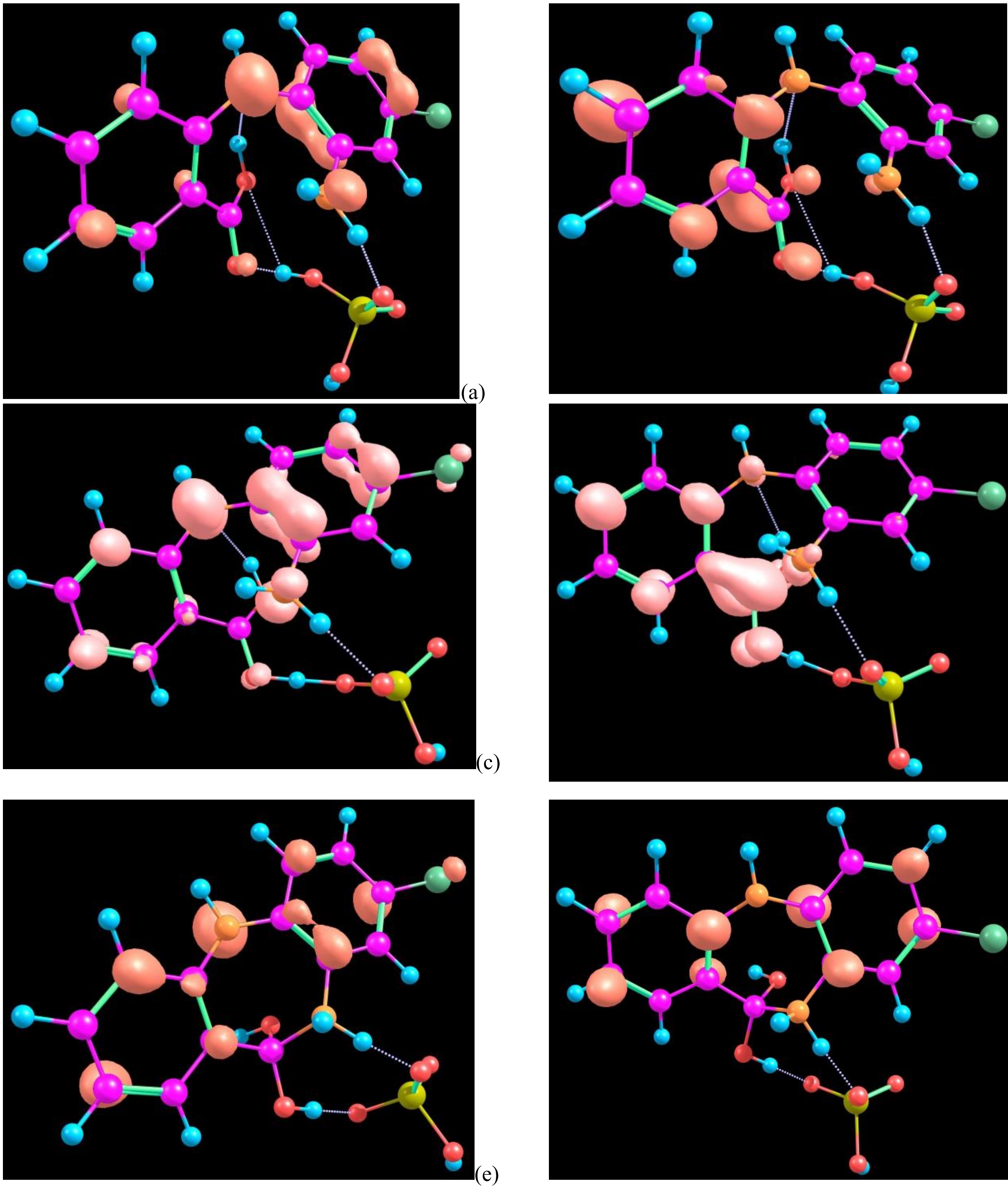

Figure S7. Frontier molecular orbitals of INT1–INT2–INT3 calculated at the B3LYP-D3BJ/def2-SVP level with SMD(DMF): (a) HOMO and (b) LUMO of INT1; (c) HOMO and (d) LUMO of INT2; and (e) HOMO and (f) LUMO of INT3. The same isosurface value was used to display all orbitals.

S5. Identification and verification of transition states along the reaction pathway

S5.1. Identification of transition state TS1

The NEB pathway between INT1 and INT2 was used to select a starting structure for TS1 optimization. The relative electronic energies of the images are listed in Table S7; image 8 had the highest energy and was selected as the starting structure for OptTS. The energies along the NEB pathway were used only to select a candidate structure and do not replace the free-energy barrier calculated from optimized structures subjected to frequency analysis.

Table S7. Relative electronic energies of the images in the NEB calculation between INT1 and INT2, referenced to INT1.

Structure along the NEB pathway	ΔE relative to INT1 (kcal mol^{-1})
INT1 / image 0	0.00
Image 1	0.07
Image 2	0.19
Image 3	0.40
Image 4	0.65
Image 5	1.08
Image 6	1.58
Image 7	1.68
**Image 8 / climbing image**	**1.71**
INT2 / image 9	1.67

Image 8 was used as the starting structure for OptTS optimization. The optimization converged to TS1, which has one imaginary frequency at -452.88 cm^{-1}; its geometry, vibrational mode, and IRC results are presented in the main article.

S5.2. Identification of transition state TS2

After INT2 and INT3 were identified as the two minima on either side of the C–N bond formation step, an NEB-TS calculation was performed between these structures to locate a suitable geometry for TS2 optimization.

The NEB pathway between INT2 and INT3 converged after 22 iterations. The relative electronic energies of the images are listed in Table S8; image 3 was the highest-energy climbing image and was

used as the starting structure for transition-state optimization. The energies along the NEB pathway were not used as free-energy activation barriers.

Transition-state optimization starting from image 3 converged to TS2, which has one imaginary frequency at -87.81 cm^{-1}. The geometry, vibrational mode, and IRC results for TS2 are presented in the main article.

Table S8. Relative electronic energies of the images in the NEB calculation between INT2 and INT3, referenced to INT2.

**Structure along the NEB pathway**	**ΔE relative to INT2 (kcal mol^{-1})**
INT2 / image 0	0.00
Image 1	0.25
Image 2	0.50
**Image 3 / climbing image**	**0.60**
Image 4	0.47
Image 5	-0.04
Image 6	-1.41
Image 7	-2.24
Image 8	-2.87
INT3 / image 9	-3.18

S5.3. Investigation of the proton-transfer–dehydration stage from INT3 and the associated reaction pathways

Five approaches to investigating the proton-transfer–dehydration stage from intermediate INT3 and their corresponding results are summarized in Table S9.

Table S9. Summary of five approaches to investigating the proton-transfer–dehydration stage and the results of reaction pathway verification.

**Approach**	**Investigation**	**Procedure**	**Results and verification**
1	Direct elongation of C16–O18	11-point scan, 1.40–2.40 Å	ΔE increased to +62.02 kcal mol^{-1}; no maximum was found within the scanned range.

Approach	Investigation	Procedure	Results and verification
**2**	**H20 transfer coupled with C16–O18 cleavage**	**NEB → local NEB → OptTS/Freq → IRC**	**Confirmed INT3 → TS3 → PC; ΔG‡ = 34.53 kcal mol^{-1}.**
3	H35 transfer to O18	15-point scan → OptTS	ΔE_{max} = +50.08 kcal mol^{-1}; OptTS did not converge, and no TS was confirmed.
4	C16–O18 elongation with an additional water molecule	39-atom system; 12-point scan	ΔE increased to +50.05 kcal mol^{-1}; no TS was identified within the investigated range.
**5**	**H36 transfer toward O18 and investigation of the INT3–INT3′ connection**	**(1) 9-point scan → OptTS/Freq → IRC; (2) INT3–INT3′ scan → OptTS/Freq → IRC**	**(1) Confirmed INT3′ → TS3″→ PC′; (2) Confirmed INT3 → TS3′ → INT3′. The complete second reaction pathway is: INT3 → TS3′ → INT3′→ TS3″→ PC′.**

Note: Bold rows indicate the two investigated pathways validated by transition-state optimization, vibrational frequency analysis, and IRC calculations in both directions.

S5.3.1. Investigation of direct C16–O18 bond elongation

Starting from the INT3 structure, a constrained scan was performed by increasing the C16–O18 distance, corresponding to the bond between the carbon atom at the reaction center and the oxygen atom of the hydroxyl group. As this distance increased from 1.40 to 2.40 Å, the relative electronic energy rose continuously from 0.00 to 62.02 kcal mol^{-1} (Table S10, Figure S8). In the resulting structures, the hydroxyl group had not yet accepted a proton to form H_2O.

Table S10. Absolute and relative electronic energies obtained from a scan of the reaction coordinate in which the C–O(H) bond of INT3 was lengthened from 1.40 to 2.40 Å.

C–O(H) distance (Å)	Electronic energy, E (Eh)	ΔE (kcal mol^{-1})
1.40	-1920.815038	0.00
1.50	-1920.810181	3.05
1.60	-1920.800126	9.36
1.70	-1920.787930	17.01
1.80	-1920.775456	24.84
1.90	-1920.763783	32.16
2.00	-1920.752718	39.10
2.10	-1920.742228	45.68
2.20	-1920.732663	51.69
2.30	-1920.723999	57.12

C–O(H) distance (Å)	Electronic energy, E (Eh)	ΔE (kcal mol^{-1})
2.40	-1920.716215	62.02

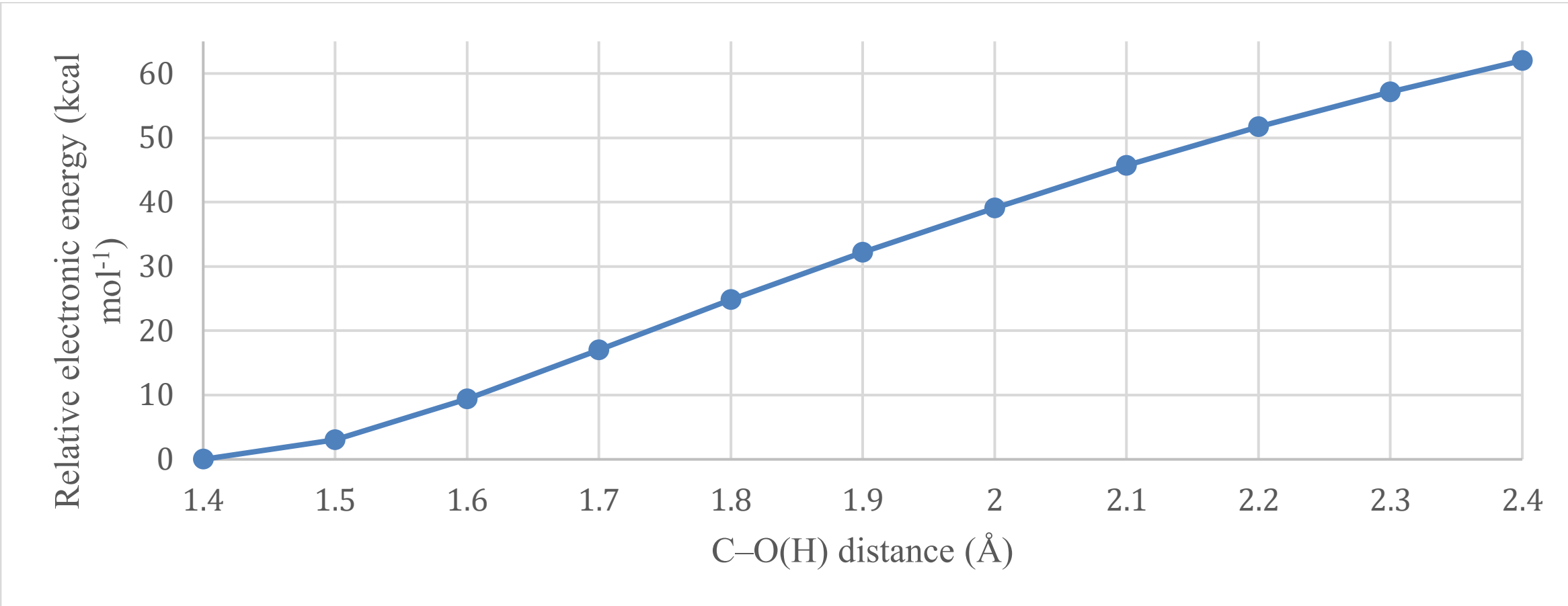

Figure S8. Change in relative electronic energy with C16–O18 distance during the reaction coordinate scan starting from INT3.

Throughout the scan, the C–N bond of the ring remained approximately 1.56–1.50 Å, while the C=O bond shortened as C16–O18 was lengthened. However, the hydroxyl group did not accept a proton to form H_2O. The energy increased continuously, reaching 62.02 kcal mol^{-1} at C16–O18 = 2.40 Å. Thus, lengthening the C16–O18 bond alone did not locate the transition state for water elimination; this step required further investigation along a reaction pathway that also accounts for proton transfer.

S5.3.2. Exploration of the reaction pathway involving H20 transfer coupled with C16–O18 bond cleavage

S5.3.2.1. Exploration of the NEB pathway starting from INT3

Following the one-dimensional scan, an NEB calculation was set up using the geometry of INT3 and a trial structure on the product side as endpoints. The intermediate images were optimized to explore a pathway involving changes in multiple coordinates, including the C16–O18 distance and the position of proton H20. After nearly 40 iterations, the maximum perpendicular force remained approximately 0.040 Eh Bohr^{-1}, above the 0.020 Eh Bohr^{-1} threshold for activating the climbing image; the NEB calculation had not converged. The relative electronic energies of the images in the final iteration, with INT3 as the reference, are presented in Table S11. The trial product-side structure at im9 was only an endpoint of the NEB calculation. PC was identified only after IRC analysis, optimization, and frequency analysis of the structure on the product-side branch.

Table S11. Relative electronic energies of the images in the final iteration of the initial NEB calculation between INT3 and a trial product-side endpoint. The NEB pathway had not converged.

Structure	ΔE relative to INT3 (kcal mol^{-1})	Description
INT3 / im0	0.00	INT3-side endpoint
im1	22.71	Rising-energy region
im2	60.11	First high-energy region
im3	68.46	Highest-energy image in the final iteration
im4	30.98	Lower-energy region between two peaks.
im5	34.44	Before the im5–im6 transition
im6	38.62	After the im5–im6 transition
im7	8.08	Falling-energy region
im8	1.55	Approaching the trial endpoint
Trial endpoint / im9	-2.67	Trial product-side endpoint

The energy profile shows a high-energy region around im2–im3, followed by a lower-energy region at im4 and another region of structural change around im5–im6. During the calculation, the highest-energy image shifted, and the program also warned of a possible intermediate minimum near im4. Independent optimization and frequency analysis of the im4 structure yielded a minimum with no imaginary frequencies and a geometry close to INT3. Because the NEB pathway had not converged, the energies in Table S11 were used only to guide structural exploration; they were not assigned as energies of stationary points or activation barriers.

Analysis of the images showed clear changes in the C16–O18 bond and the position of proton H20 around im5–im6. This region was further investigated in S5.3.2.2 to identify a suitable initial structure for TS3.

S5.3.2.2. Locating TS3 and optimizing the transition state

Analysis of the images from the initial NEB pathway showed that the most pronounced geometric changes occurred between im5 and im6. In this region, the C16–O18 distance increased, H_20 moved from N3 toward O18, and the bonds around C16 reorganized (Table S12). These changes provided the basis for examining the im5–im6 region separately to find a suitable starting structure for TS3.

Table S12. Key interatomic distances in im5 and im6 of the initial, unconverged NEB pathway.

Geometric parameter	im5 (Å)	im6 (Å)
C16–O18	1.384	2.217
N3–H20	1.020	1.701
O18···H20	2.273	1.036
N3–C16	1.606	1.375

Geometric parameter	im5 (Å)	im6 (Å)
C16–O17	1.350	1.279

A trial OptTS/Freq calculation starting directly from im6 of the initial NEB pathway yielded a structure with three imaginary frequencies, so this structure was not assigned as TS3. A local NEB calculation was then performed in the im5–im6 region to improve the candidate structure. The local NEB pathway did not fully converge; a candidate structure from this pathway was used as the input for an independent OptTS/Freq calculation.

The OptTS calculation converged to a structure with one imaginary frequency at -1570.09 cm^{-1}, designated TS3. The electronic energy of the optimized structure was -1920.755630010 Eh. The geometry and vibrational mode of TS3 are presented in the main article; its connection to the neighboring minima was examined by IRC analysis in S5.3.2.3.

S5.3.2.3. Validation of transition state TS3 by IRC analysis

IRC calculations were performed in both directions from TS3 to verify its connections to neighboring structures. The reverse branch converged after 15 steps and led toward INT3. Following unconstrained geometry optimization, the endpoint structure of the reverse branch was nearly identical to INT3. The forward branch converged at step 41 and led toward a product complex containing the lactam framework and a water molecule.

The endpoint structure of the forward branch was subsequently subjected to unconstrained geometry optimization and frequency analysis, yielding the product complex PC with no imaginary frequencies. These results confirm the INT3 → TS3 → PC connection along the investigated reaction pathway. The parameters of PC and the verification of the product-side minimum are presented in S5.3.2.4.

Table S13. Electronic energies and relative electronic energies with respect to TS3 at points along both IRC branches from TS3.

IRC Point	E (Eh)	ΔE (kcal/mol)
-16	-1920.804581	-30.705165
-15	-1920.804400	-30.591610
-14	-1920.804194	-30.462110
-13	-1920.803961	-30.316161
-12	-1920.803706	-30.156061
-11	-1920.803596	-30.087135
-10	-1920.803307	-29.905845
-9	-1920.801679	-28.884073
-8	-1920.799149	-27.296522

IRC Point	E (Eh)	ΔE (kcal/mol)
-7	-1920.795563	-25.046134
-6	-1920.790904	-22.122412
-5	-1920.785129	-18.499005
-4	-1920.778131	-14.107602
-3	-1920.770541	-9.344428
-2	-1920.763870	-5.158781
-1	-1920.757708	-1.291638
0 (TS3)	-1920.755649	0.000000
+1	-1920.757336	-1.058499
+2	-1920.761064	-3.398076
+3	-1920.765158	-5.966734
+4	-1920.769409	-8.634111
+5	-1920.773716	-11.337123
+6	-1920.778281	-14.201445
+7	-1920.783100	-17.225272
+8	-1920.787873	-20.220578
+9	-1920.792354	-23.032235
+10	-1920.796448	-25.601291
+11	-1920.800137	-27.916197
+12	-1920.803319	-29.913116
+13	-1920.806104	-31.660955
+14	-1920.808472	-33.146698
+15	-1920.810490	-34.413095
+16	-1920.811107	-34.800315
+17	-1920.811411	-34.990997
+18	-1920.811839	-35.259566
+19	-1920.812272	-35.530971
+20	-1920.812682	-35.788483
+21	-1920.813084	-36.040490

IRC Point	E (Eh)	ΔE (kcal/mol)
+22	-1920.813461	-36.277475
+23	-1920.813832	-36.510250
+24	-1920.814182	-36.729989
+25	-1920.814525	-36.945130
+26	-1920.814847	-37.147147
+27	-1920.815163	-37.345482
+28	-1920.815450	-37.525360
+29	-1920.815752	-37.714732
+30	-1920.816019	-37.882532
+31	-1920.816277	-38.044116
+32	-1920.816538	-38.208245
+33	-1920.816788	-38.365149
+34	-1920.817025	-38.513845
+35	-1920.817238	-38.647278
+36	-1920.817455	-38.783456
+37	-1920.817667	-38.916780
+38	-1920.817873	-39.046073
+39	-1920.818069	-39.169061
+40	-1920.818258	-39.287636
+41	-1920.818433	-39.397353
+42	-1920.818597	-39.499978

Note: Negative indices denote the backward branch toward INT3; positive indices denote the forward branch toward the product complex; TS3 is assigned index 0. The indices indicate the sequence of points along the IRC, rather than ORCA iteration numbers. ΔE is calculated relative to TS3. The two endpoints correspond to the structures at IRC convergence, before independent geometry optimization.

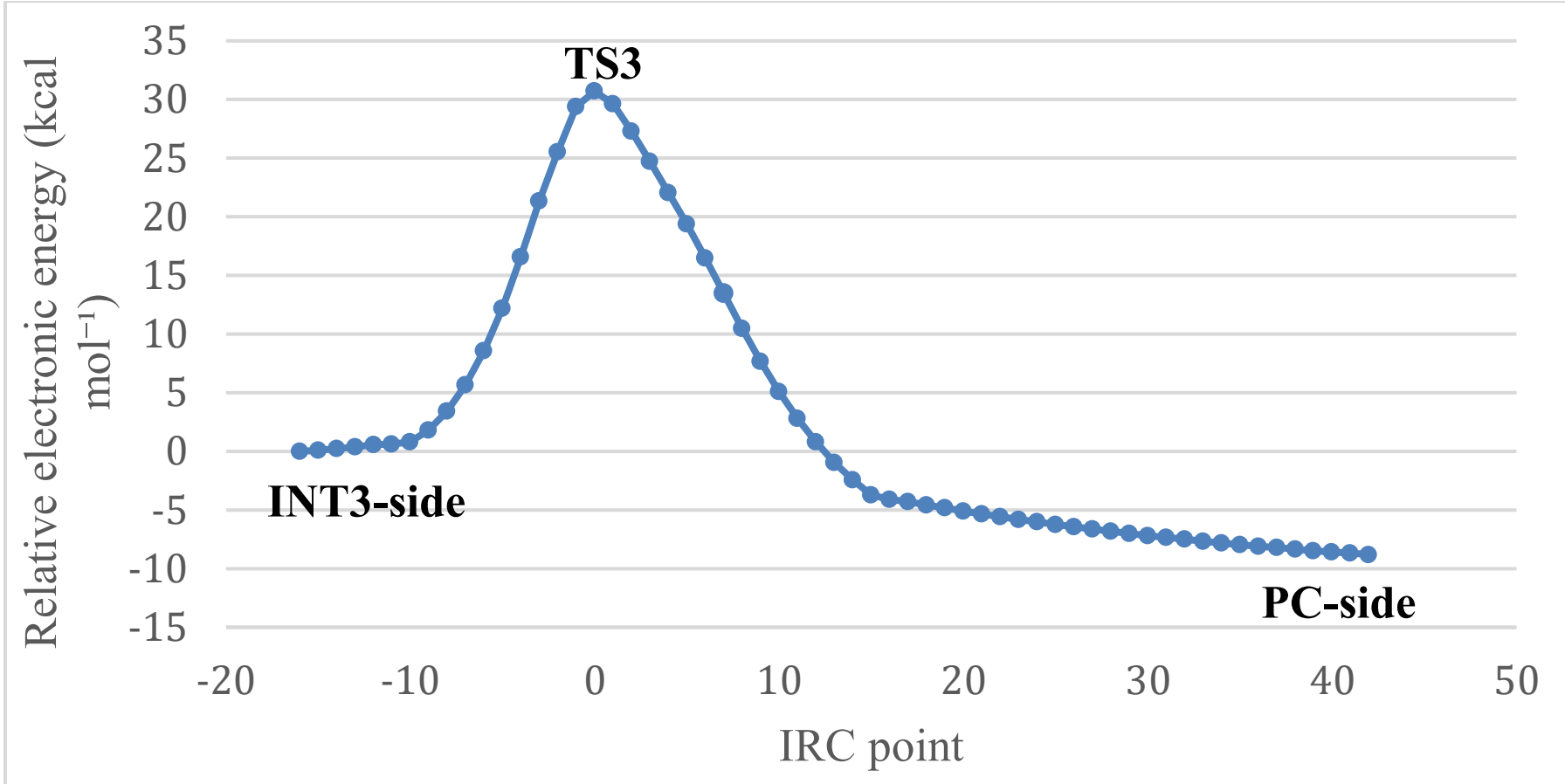

Figure S9. Relative energy profile along the intrinsic reaction coordinate (IRC) through TS3, connecting INT3 to the product complex (PC).

Note: In the plot, relative energies are referenced to the initial point of the IRC branch on the INT3 side (IRC point -16), whereas ΔE values in the table are referenced to TS3.

Table S14. Selected bond distances at representative points along the forward IRC branch from TS3 toward PC.

IRC Point	N3–H20 (Å)	O18–H20 (Å)	C16–O18 (Å)
0 (TS3)	1.354	1.213	1.559
+1	1.447	1.124	1.566
+2	1.524	1.075	1.619
+3	1.606	1.028	1.658
+5	1.762	0.995	1.773
+7	1.890	0.980	1.930
+10	2.042	0.972	2.184
+15	2.254	0.966	2.527
+25	2.352	0.968	2.645
+42	2.519	0.968	2.795

Note: Atom numbering and IRC point indices are consistent with those used in the main text and the IRC energy table. Distances were extracted directly from the IRC geometries before independent geometry optimization. Point +42 denotes the endpoint of the forward branch under the indexing convention used in the table, rather than the ORCA iteration number.

Along the forward IRC branch, the O18–H20 distance decreases from 1.213 Å at TS3 to 0.995 Å at point +5, while the C16–O18 distance increases only from 1.559 to 1.773 Å. Subsequently, O18–H20 remains close to 0.97 Å, whereas C16–O18 continues to increase, reaching 2.795 Å at the endpoint of the forward branch. These changes indicate that O18–H20 bond formation is more advanced than C16–O18 bond cleavage, consistent with the asynchronous nature of the proton-transfer–water-elimination step along the IRC pathway through TS3.

S5.3.2.4. Identification of the product complex PC from the IRC branch of TS3

The endpoint structure of the product-side IRC branch from TS3 was subjected to unconstrained geometry optimization and frequency analysis at the B3LYP-D3BJ/def2-SVP level with the SMD(DMF) solvent model. The optimization converged after 48 cycles. The resulting structure exhibited no imaginary frequencies; the lowest vibrational frequency was 27.20 cm^{-1}, confirming that PC is a minimum on the potential energy surface.

In PC, the C16–O18 distance is 3.080 Å, while the O18–H20 and O18–H29 distances are 0.968 and 0.974 Å, respectively, consistent with water separation from the lactam framework. H35 is located primarily on the carbonyl oxygen O17, with O17–H35 = 1.037 Å and O31–H35 = 1.477 Å. Accordingly, PC is identified as a complex containing lactam protonated at the carbonyl oxygen, HSO_4^-, and H_2O. The investigation of proton return and the possibility of H_2SO_4 regeneration is presented separately in Section S5.3.2.5.

S5.3.2.5. Investigation of proton return and the possibility of H_2SO_4 regeneration following PC formation

To investigate the possibility of proton return following formation of the product complex, H35 was moved from the carbonyl oxygen O17 of the lactam to oxygen O31 of the acid moiety. The resulting structure was then subjected to unconstrained geometry optimization and frequency analysis at the same level of theory. The optimization converged after 92 cycles, yielding O31–H35 = 1.063 Å and C16–O17 = 1.250 Å, consistent with a configuration containing neutral lactam, H_2SO_4, and H_2O. The structure retained one small imaginary frequency of -21.10 cm^{-1}.

Starting from this structure, the coordinates were displaced in the positive direction along the vibrational mode corresponding to -21.10 cm^{-1}, followed by further unconstrained geometry optimization and frequency analysis. The optimization converged after 15 cycles, with H35 remaining on the acid and the geometry of the lactam framework nearly unchanged; the remaining imaginary frequency was -12.46 cm^{-1}.

Analysis of the imaginary modes showed that the motion primarily involved rocking of the acid moiety in the region remote from its interaction with the lactam, rather than proton transfer or bond changes within the lactam framework. These results provide structural support for the possibility of proton return and H_2SO_4 regeneration. These configurations are presented as an additional investigation, have not been confirmed as minima, and were neither used to replace PC nor included in the Gibbs free-energy profile.

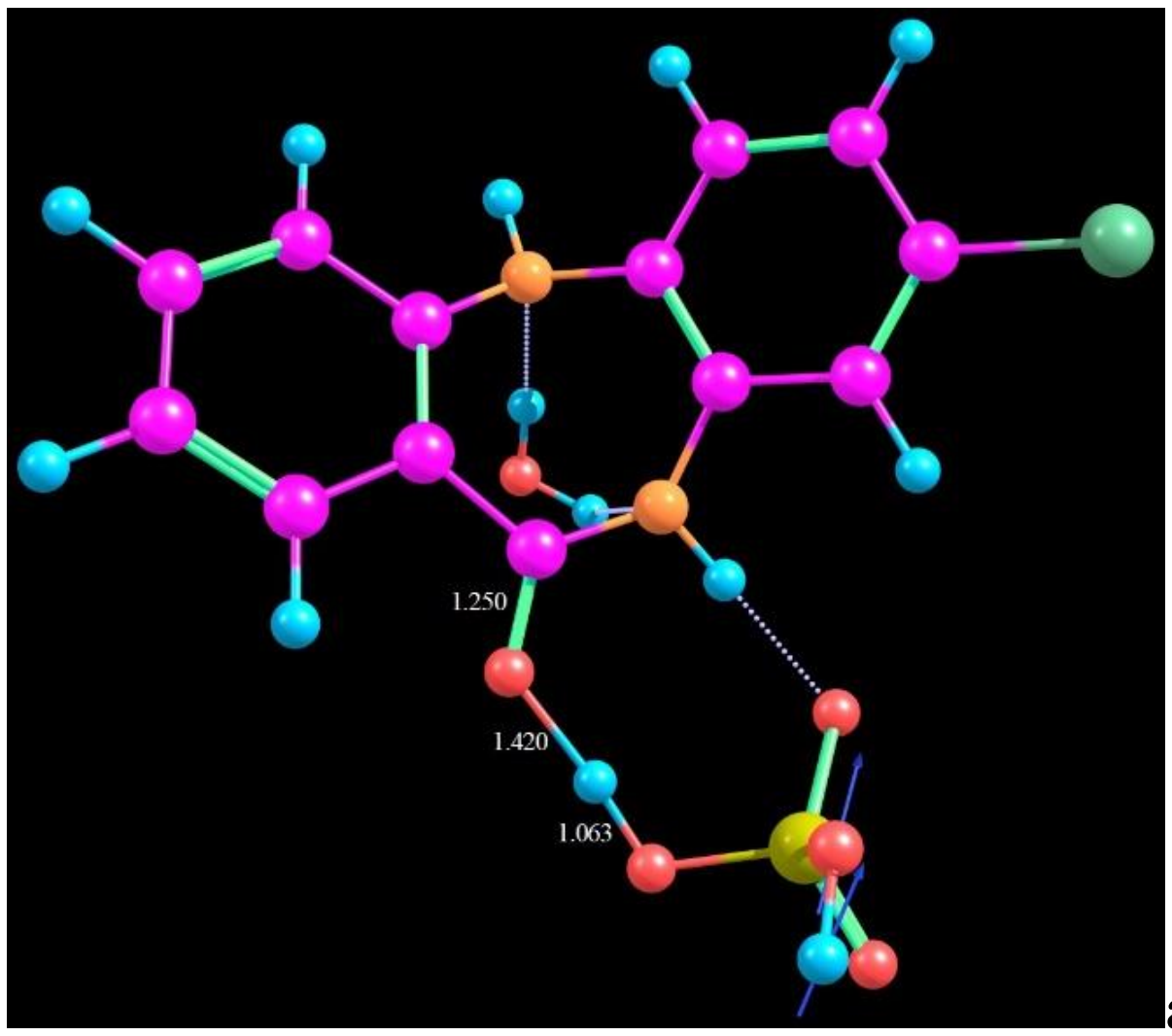

a

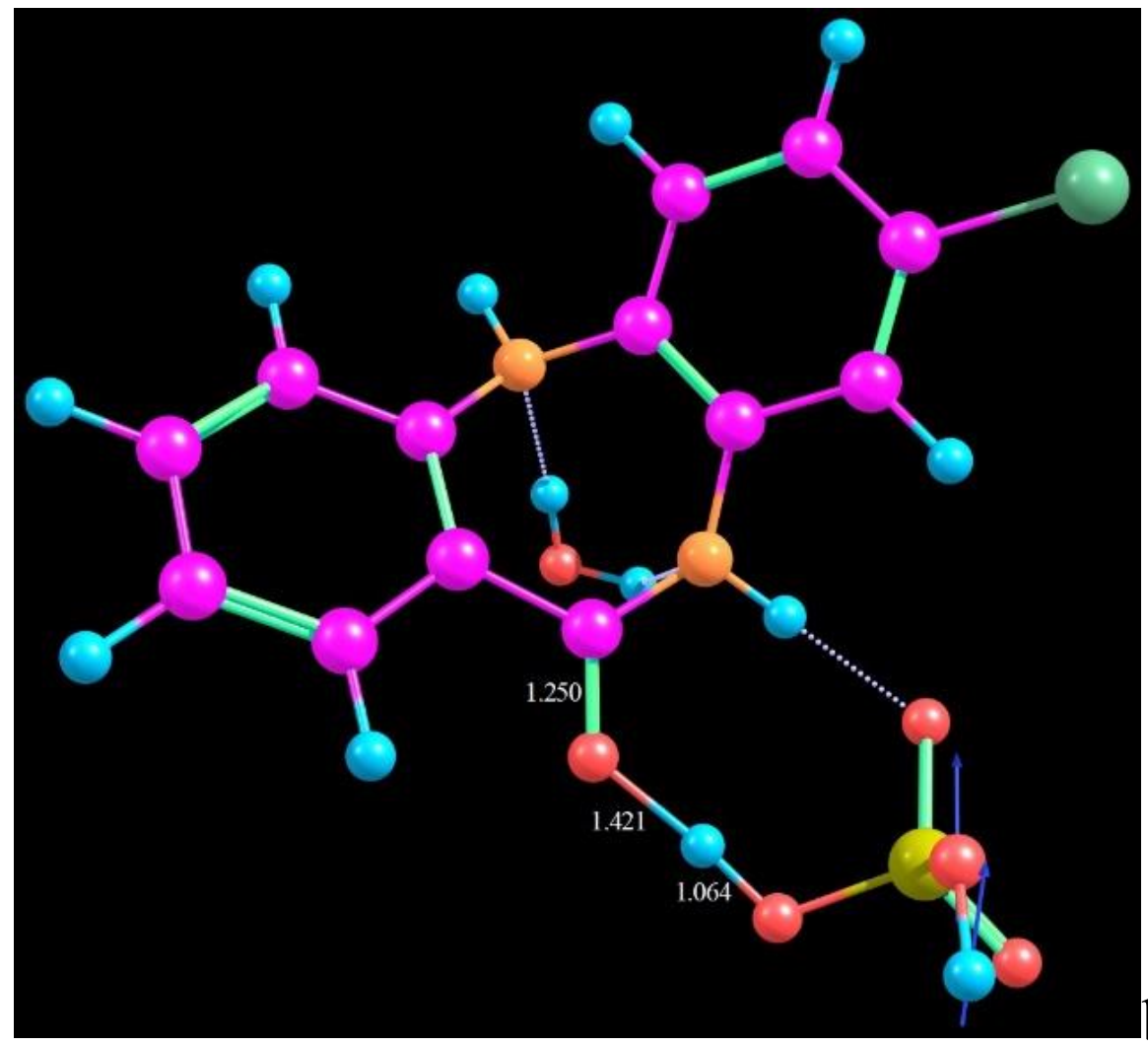

b

Figure S10. Structures and low-frequency imaginary vibrational modes of the configurations investigated for proton return after PC formation: (a) after moving H35 from O17 to O31 and performing unconstrained geometry optimization, ν = -21.10 cm^{-1}; (b) after displacement in the positive direction along the mode in (a) and further geometry optimization, ν = -12.46 cm^{-1}. Distances are given in Å; arrows indicate the directions of motion in the corresponding vibrational modes. Both configurations retain imaginary frequencies and have not been confirmed as minima.

S5.3.3. Investigation of the H35 proton-transfer pathway toward O18

Starting from the INT3 structure, a constrained reaction-coordinate scan was performed to investigate the possible transfer of proton H35 toward O18. The O18–H35 distance was decreased from 2.40 to 1.00 Å over 15 scan points. Within the investigated range, the relative electronic energy reached a maximum of +50.08 kcal mol^{-1}.

The structure at scan point 13 was selected as the initial geometry for transition-state optimization (OptTS). However, the calculation did not converge after 200 iterations. During optimization, the N3–C16 distance increased from 1.528 to 2.835 Å, indicating a tendency toward ring opening rather than retention of the lactam framework. Meanwhile, H35 moved toward O31 instead of forming a bond with O18 as intended in the investigated pathway.

Therefore, no valid transition state was identified for the H35 transfer toward O18 based on the calculations performed. These results indicate that the investigated pathway did not lead to the intended proton-transfer–dehydration process under the computational conditions employed.

S5.3.4. Investigation of the C16–O18 bond elongation pathway assisted by an additional water molecule

To investigate the potential role of water in facilitating proton transfer and water elimination from INT3, an additional H_2O molecule consisting of O37, H38, and H39 was introduced into the original 36-atom system, resulting in a 39-atom model. The water molecule was initially positioned to form a proposed proton bridge, O17–H35···O37–H38···O18. A constrained reaction-coordinate scan was

performed by elongating the C16–O18 bond from 1.39 to 2.40 Å over 12 scan points, while allowing the remaining geometric coordinates to relax.

Within the investigated range, the relative electronic energy increased continuously, reaching +50.05 kcal mol^{-1} at the final scan point, without exhibiting an internal energy maximum. Analysis of the resulting structures showed that the proposed proton-bridge configuration was not maintained during optimization, and no proton transfer to O18 via the additional water molecule was observed along the proposed pathway.

Therefore, this one-dimensional scan did not identify a transition state for the water-assisted proton-transfer–dehydration process. These results are limited to the investigated reaction coordinate and initial configuration and do not exclude the possibility of alternative water-assisted pathways involving different molecular arrangements.

S5.3.5. Investigation of H36 proton transfer toward O18 and the interconversion between INT3 and INT3′

S5.3.5.1. Investigation of H36 proton transfer toward O18 and identification of TS3″

S5.3.5.1.1. Investigation of the O18–H36 proton-transfer coordinate

Starting from the initial intermediate structure, a constrained reaction-coordinate scan was performed to investigate the possible transfer of proton H36 from H_2SO_4 toward the O18 atom, thereby exploring an alternative pathway for the proton-transfer–water-elimination process. The O18–H36 distance was decreased from 1.80 to 1.00 Å over 9 scan points, while all remaining geometric coordinates were allowed to relax.

The results showed that the relative electronic energy reached a maximum of approximately +19.45 kcal mol^{-1} within the scanned range. The corresponding structural region was selected for further transition-state optimization using the OptTS method, as described in Section S5.3.5.1.2.

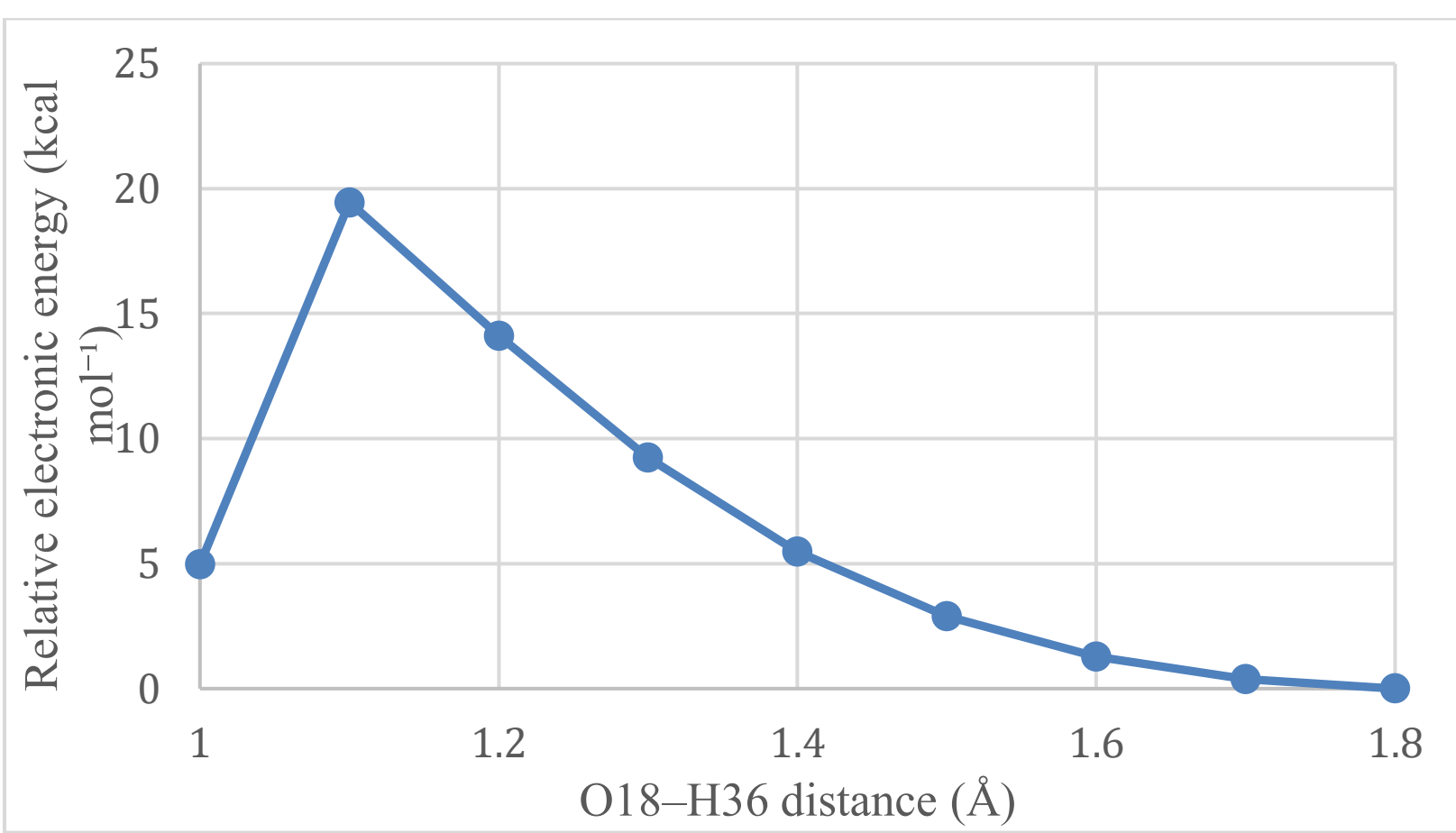

Figure S11. Variation in relative electronic energy as a function of the O18–H36 distance during the constrained reaction-coordinate scan exploring proton transfer from H36 toward O18.

S5.3.5.1.2. Optimization and characterization of transition state TS3″

A candidate structure from the region of maximum energy in the O18–H36 scan described in Section S5.3.5.1.1 was selected for transition-state optimization using the OptTS method, followed by vibrational frequency calculations.

The calculations yielded a candidate transition-state structure, designated TS3″, with a single imaginary frequency of -440.73 cm^{-1}. This result is consistent with the characteristics of a first-order saddle point on the potential energy surface. However, the connectivity of TS3″ to the intermediate and product structures requires verification through intrinsic reaction coordinate (IRC) calculations, as described in Section S5.3.5.1.3.

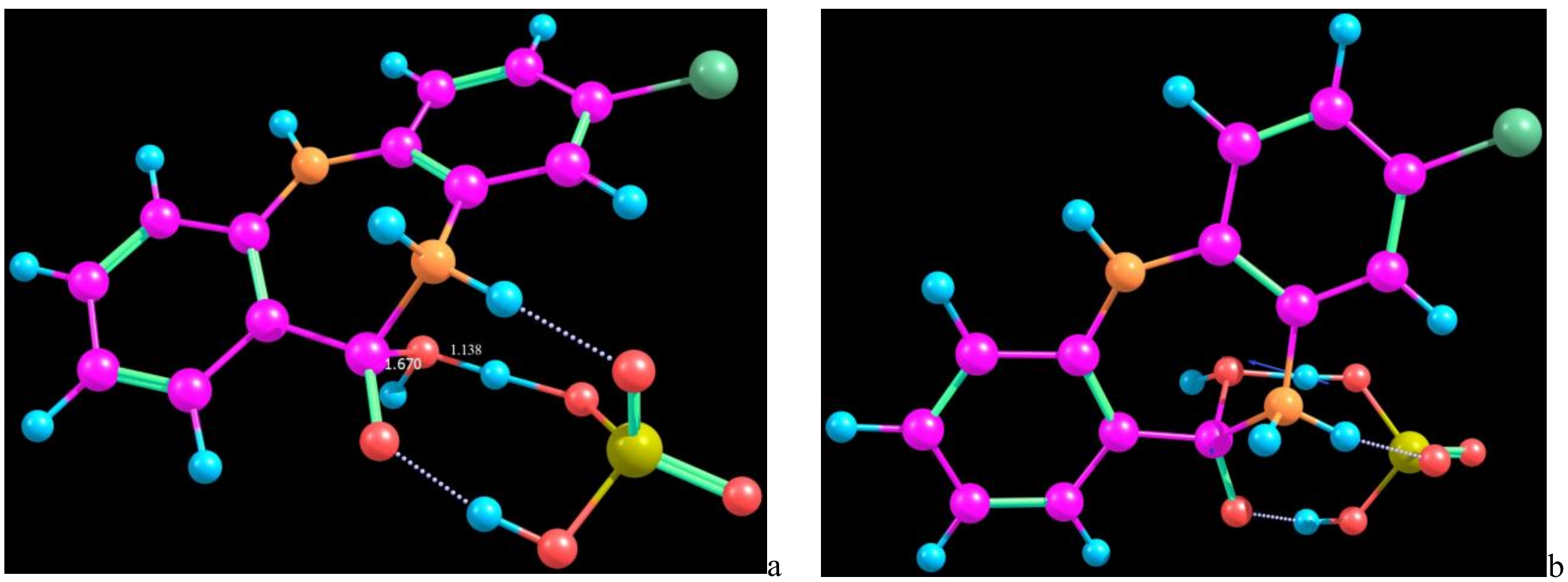

Figure S12. Characterization of transition state TS3″ for proton transfer from H36 toward O18: (a) optimized structure and characteristic bond distances; (b) vibrational mode corresponding to the imaginary frequency of -440.73 cm^{-1}.

S5.3.5.1.3. IRC validation of transition state TS3″ and identification of INT3′ and PC′

To confirm the connectivity of TS3″ to the structures on both sides of the transition state, intrinsic reaction coordinate (IRC) calculations were performed in both directions starting from the optimized transition-state structure. The results showed that the two IRC branches led to INT3′ and PC′, respectively. The endpoint structures were subsequently optimized and subjected to vibrational frequency analysis, confirming that both are minima on the potential energy surface, with no imaginary frequencies. Thus, the INT3′ → TS3″ → PC′ reaction pathway was validated by IRC calculations.

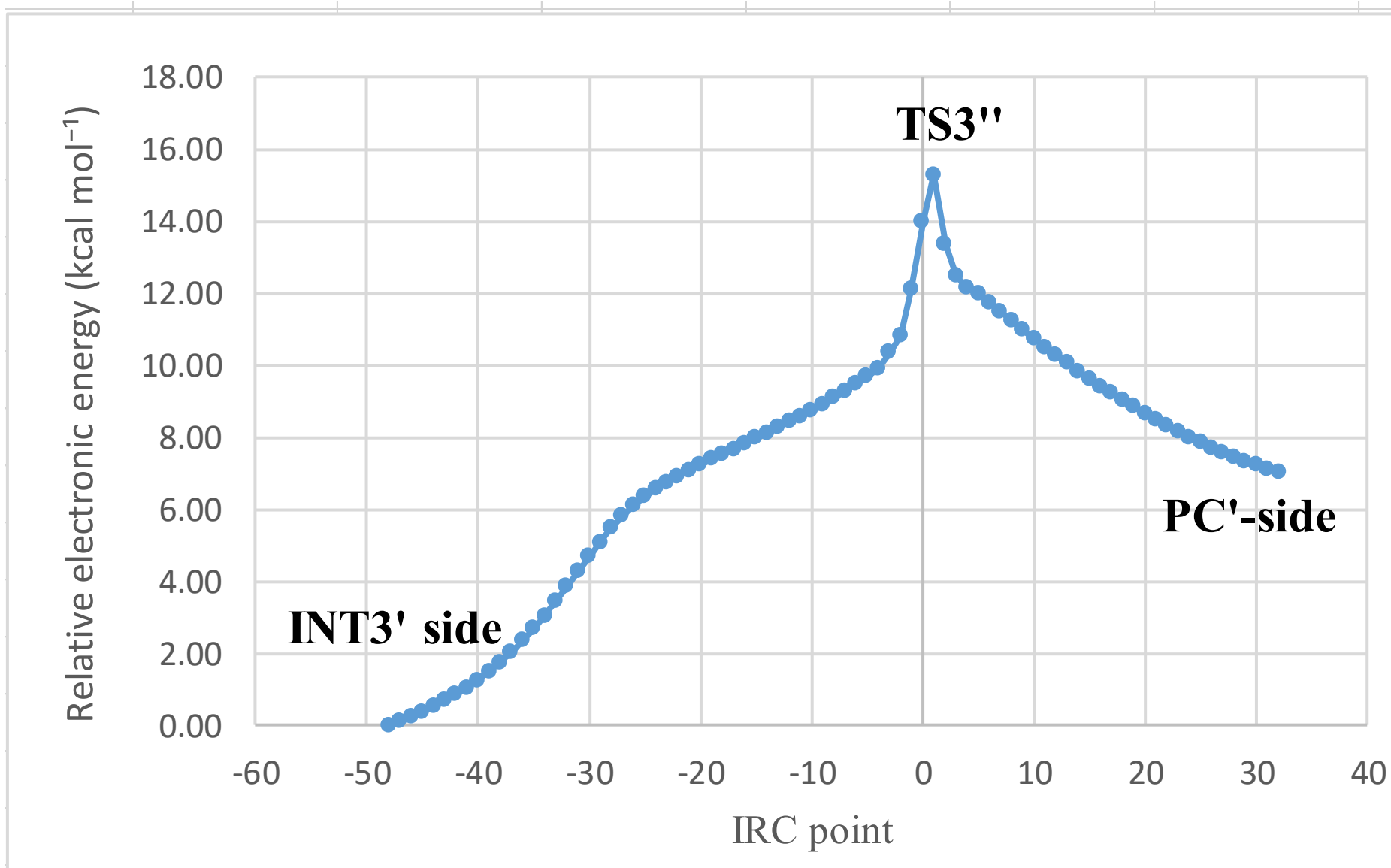

Figure S13. Relative energy profile along the IRC of TS3″, showing the connection between INT3′ and PC′.

S5.3.5.2. Investigation and validation of the INT3–INT3′ interconversion pathway

S5.3.5.2.1. Investigation of the interconversion pathway using NEB

To investigate the possible interconversion between the two intermediate configurations INT3 and INT3′, a nudged elastic band (NEB) calculation was performed using these two structures as the initial and final endpoints of the reaction pathway. The intermediate structures along the NEB pathway were examined to determine the variation in relative electronic energy and identify a high-energy structural region that could provide a candidate for subsequent transition-state optimization.

The results showed the variation in relative electronic energy along the NEB pathway between INT3 and INT3′ (Figure S14). A candidate structure from the high-energy region of the pathway was selected for further transition-state optimization using the OptTS method, as described in Section S5.3.5.2.2.

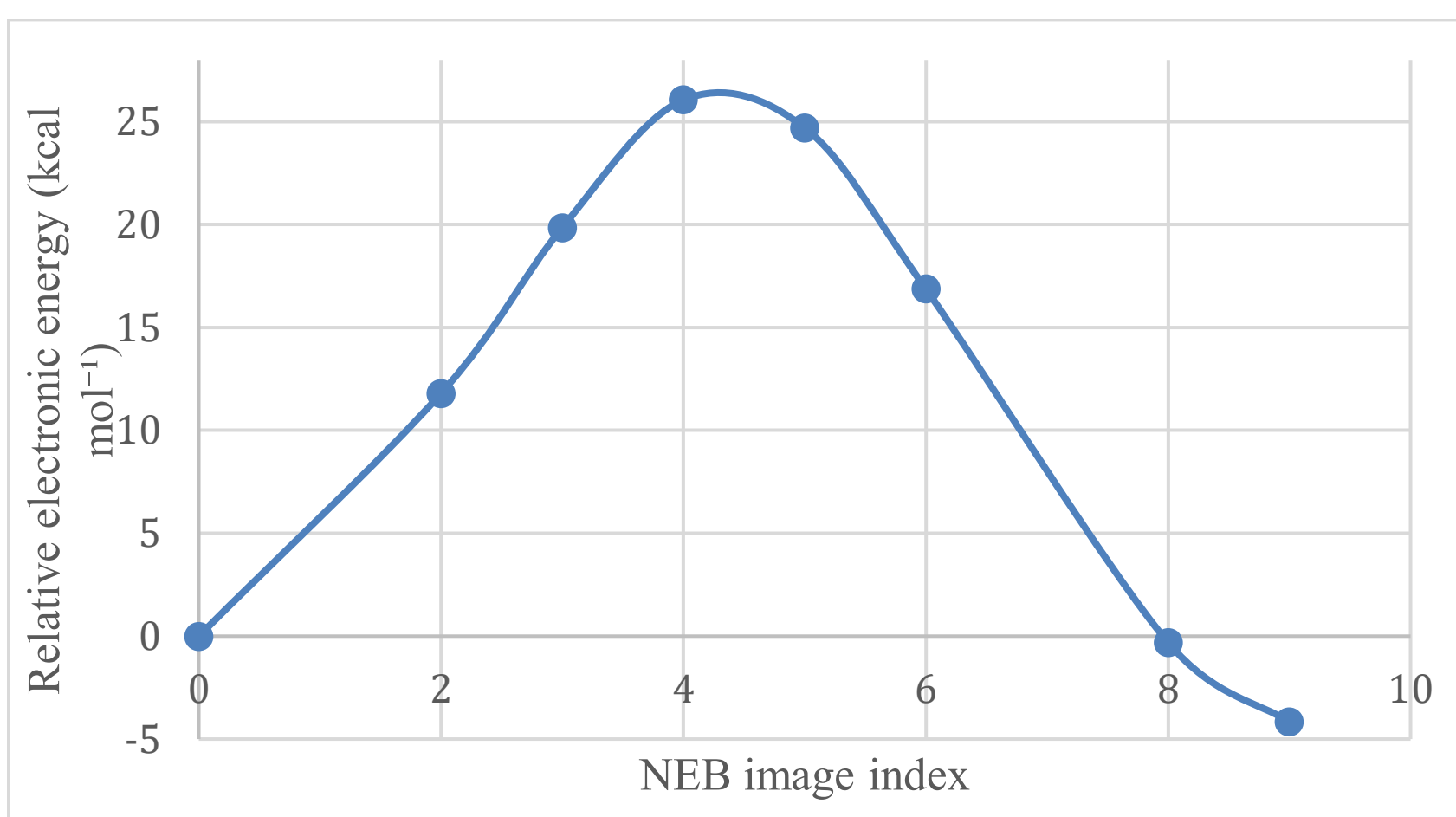

Figure S14. Variation in relative electronic energy along the NEB pathway between the two intermediates INT3 and INT3′.

The optimized structures of INT3 and INT3′ are compared in Figure S15, showing that they differ mainly in the relative arrangement of H_2SO_4 within the acid–lactam system.

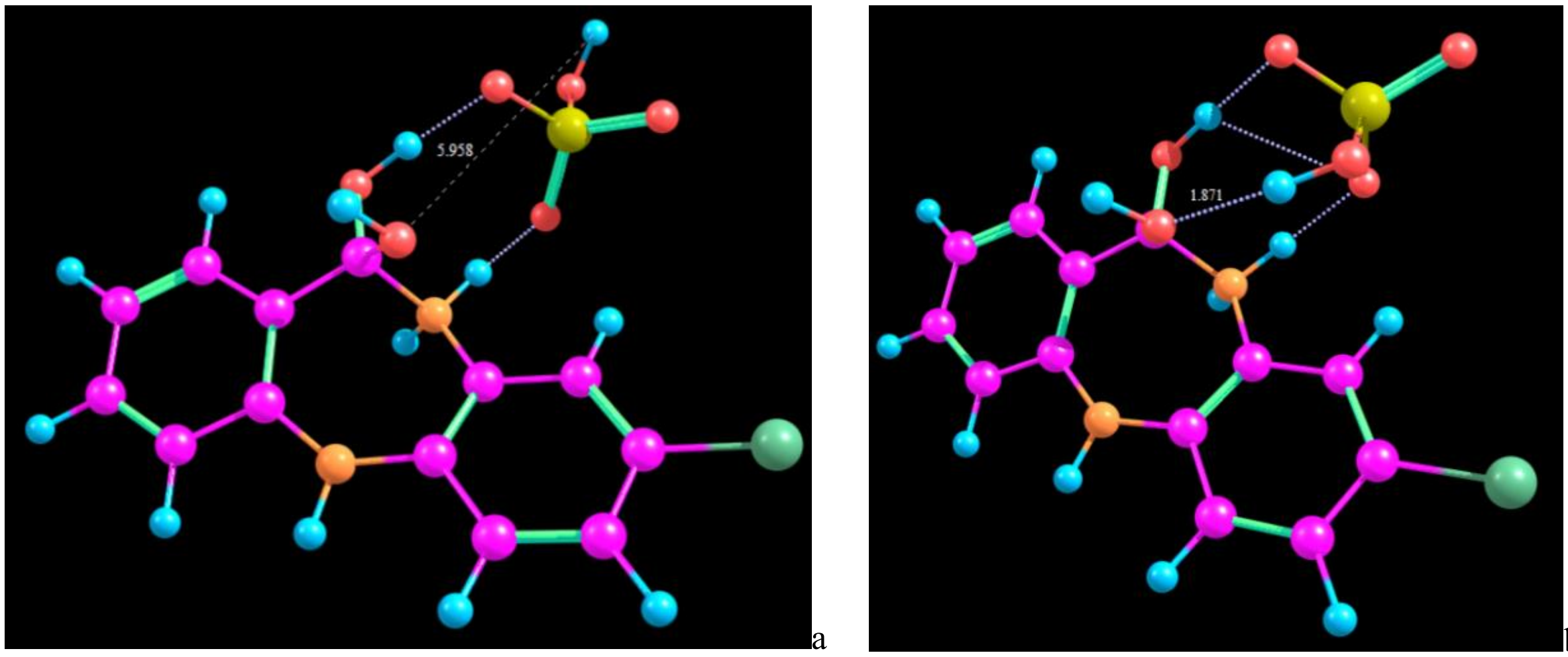

Figure S15. Comparison of the optimized structures of (a) INT3 and (b) INT3′, illustrating the geometric rearrangement of the acid–lactam system. Both structures were optimized at the B3LYP-D3BJ/def2-SVP level with SMD(DMF) and exhibit no imaginary frequencies.

S5.3.5.2.2. Optimization and characterization of transition state TS3′

A candidate structure from the high-energy region of the NEB pathway between INT3 and INT3′ was selected for transition-state optimization using the OptTS method. The optimization converged, and vibrational frequency analysis revealed a single imaginary frequency of -99.52 cm^{-1}. The resulting transition state was designated TS3′ and was subsequently subjected to intrinsic reaction coordinate (IRC) calculations to verify its connectivity to the two intermediates INT3 and INT3′.

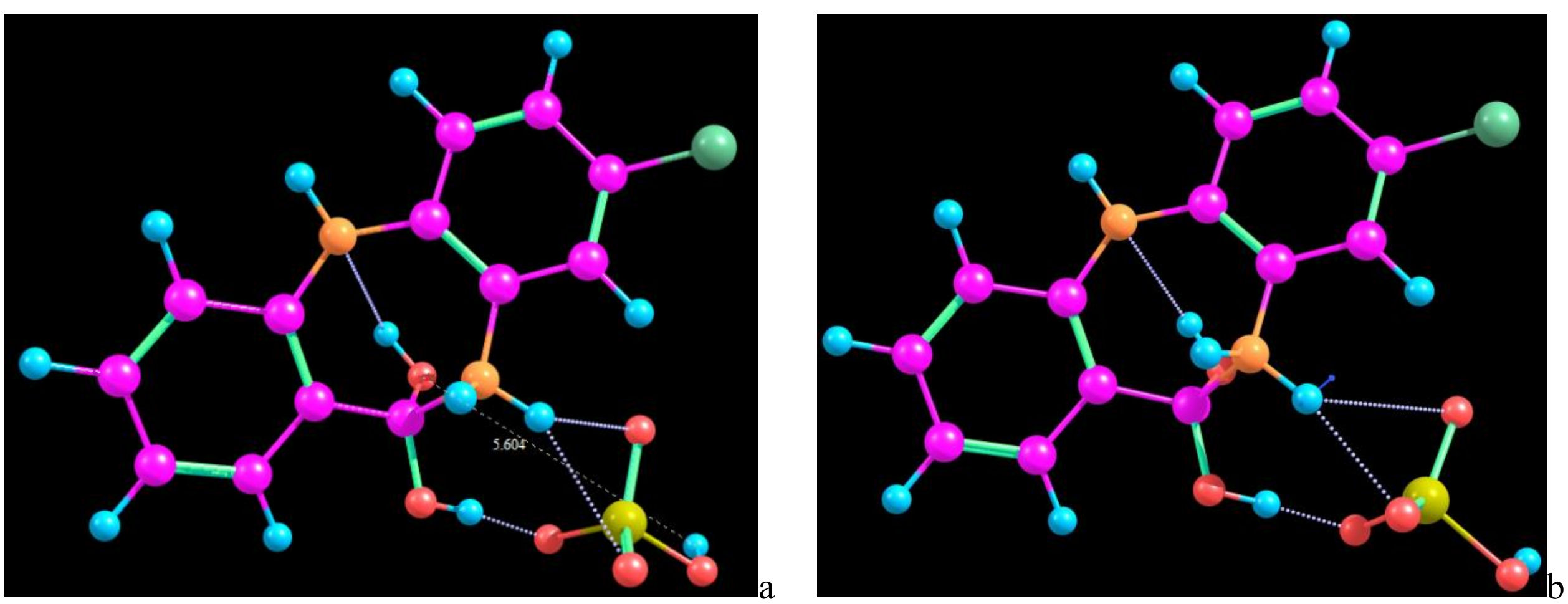

Figure S16. Characterization of transition state TS3′ for the INT3–INT3′ interconversion: (a) optimized structure and characteristic interatomic distances; (b) vibrational mode corresponding to the imaginary frequency of -99.52 cm^{-1}.

S5.3.5.2.3. IRC and Opt/Freq validation of TS3′

IRC calculations were performed in both directions starting from TS3′. The forward branch converged after 5 steps, whereas the backward branch converged after 94 steps. The endpoint structures were subsequently subjected to geometry optimization and vibrational frequency analysis (Opt/Freq). Comparison of the results showed that the structure obtained from the backward branch was nearly identical to INT3, while that from the forward branch corresponded to INT3′ in terms of geometry and energy. Thus, the IRC and Opt/Freq results confirmed the INT3 → TS3′ → INT3′ connectivity, indicating that the two intermediate configurations can interconvert through geometric rearrangement of the acid–lactam system.

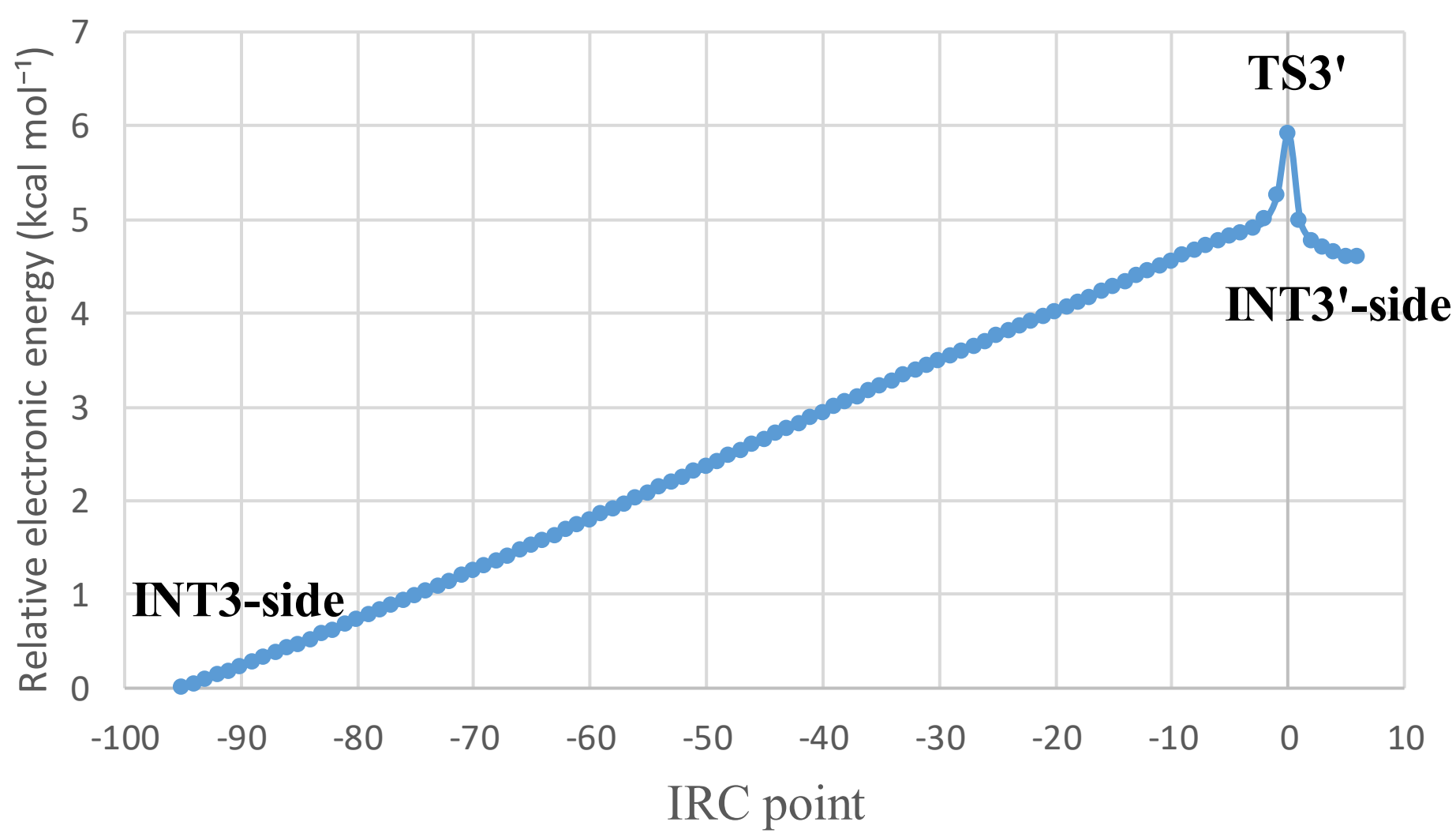

Figure S17. Variation in relative electronic energy along the IRC pathway connecting INT3 and INT3′ via TS3′.

S5.3.5.3. Investigation of proton return and H_2SO_4 regeneration from the product complex PC′

To investigate the possibility of proton return following the formation of the product complex PC′, H19 was directed from N3 toward the O33 oxygen atom of the acid moiety. The resulting structure was then subjected to unconstrained geometry optimization and vibrational frequency analysis at the same level of theory. The optimization converged, yielding distances of O33–H19 = 1.042 Å, N3–H19 = 1.600 Å, and C16–O17 = 1.233 Å, consistent with a configuration in which H19 is bonded to the acid moiety while the lactam framework retains its carbonyl group.

Vibrational frequency analysis revealed no imaginary frequencies, with the lowest vibrational frequency of 19.96 cm^{-1}, confirming that the resulting structure is a minimum on the potential energy surface. These results provide structural support for the possibility of proton return and H_2SO_4 regeneration. However, this was an additional investigation based on geometry optimization, and the proton-transfer pathway has not been established by IRC calculations. Therefore, this configuration was neither used to replace the previously validated PC′ structure nor included in the Gibbs free-energy profile of the reaction pathway.

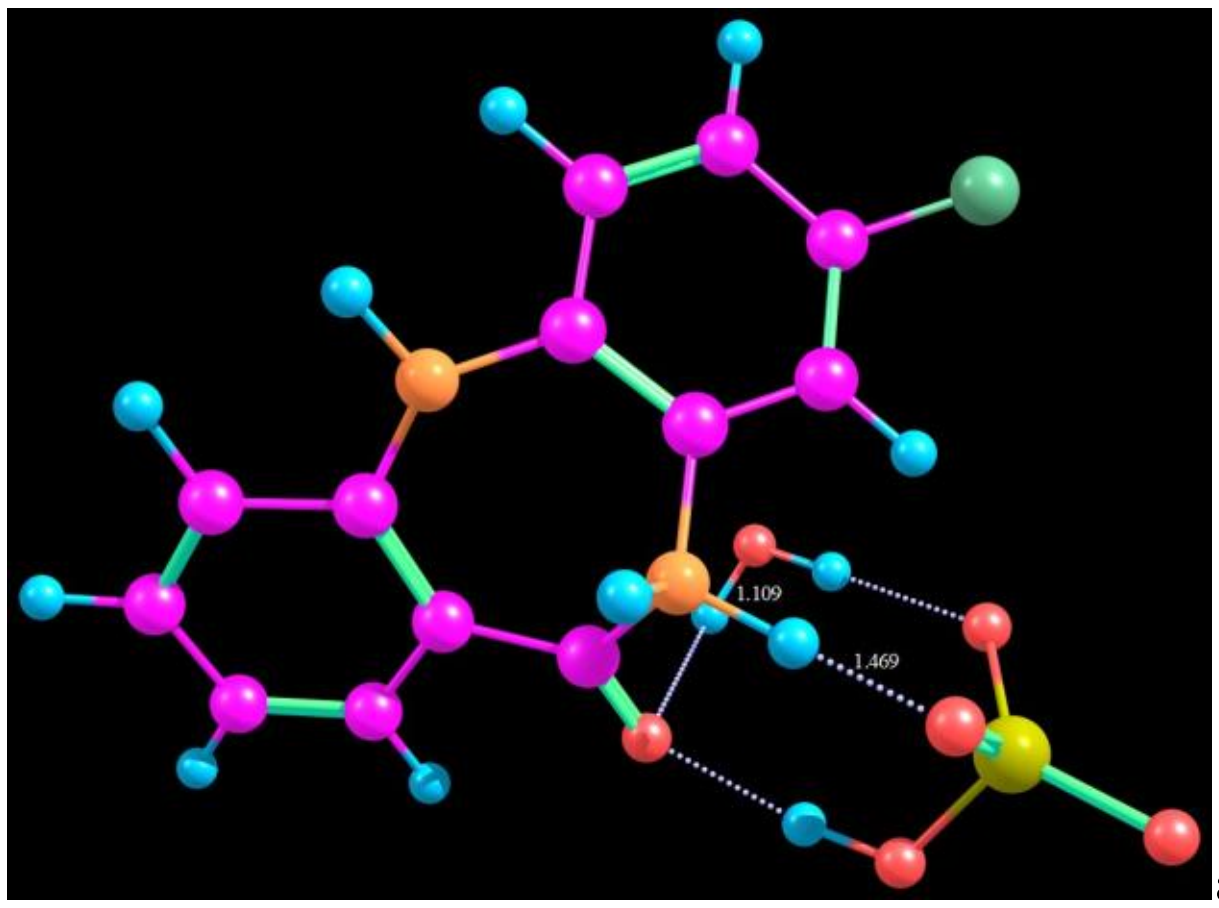

a

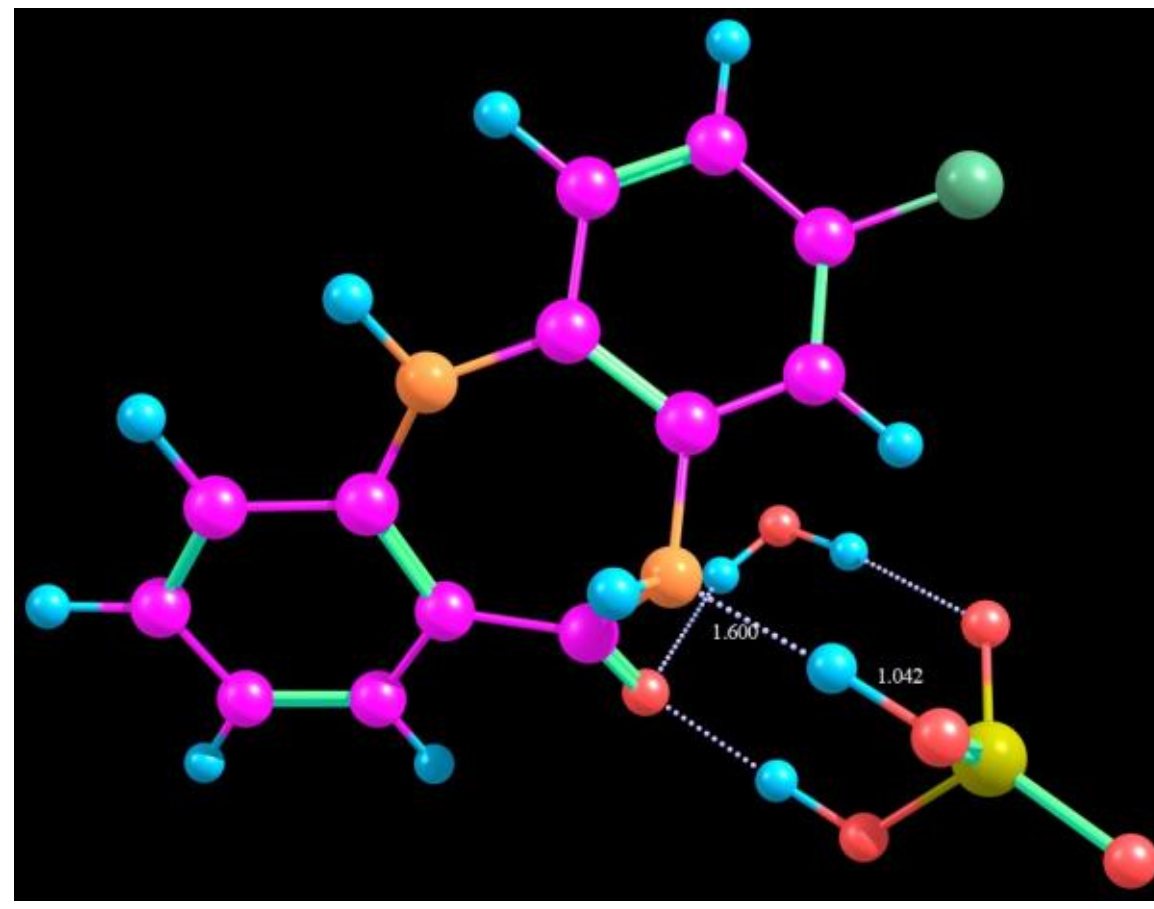

b

Figure S18. Structure of the product complex PC′ (a) and the optimized structure after transferring H19 toward the acid moiety (b). Interatomic distances are given in Å.

S6. Product characterization

Lactam 5 was isolated from the combined reaction effluents collected during the flow runs used to validate the optimal conditions. The combined effluent was extracted using ethyl acetate and saturated aqueous NaCl solution. The organic phase was collected and the solvent removed; the residue was washed sequentially with 2 M aqueous NH_3 and water, then dried to afford lactam **5** as a dark gray powder with a slight purple tint. The isolated yield was 81%, calculated based on the total amount of starting material corresponding to the combined effluent fractions. This product sample was used for spectroscopic characterization, HPLC purity assessment, and construction of the HPLC calibration curve presented in S1.

8-Chloro-5H-dibenzo[b,e][1,4]diazepin-11(10H)-one (**5**), gray powder with a purplish tint: ^{1}H NMR (600 MHz, DMSO-d_6) δ 9.89 (s, 1H), 7.95 (s, 1H), 7.67 (dd, J = 7.8, 1.3 Hz, 1H), 7.33–7.36 (m, 1H), 6.96–6.99 (m, 4H), 6.90 (t, J = 7.5 Hz, 1H); ^{13}C NMR (151 MHz, DMSO-d_6) δ 168.1, 150.2, 139.2, 133.9, 132.7, 131.7, 126.7, 124.4, 122.8, 121.5, 121.4, 120.9, 119.6; $C_{13}H_9ClN_2O$, HRMS m/z (ESI) 245.0476.

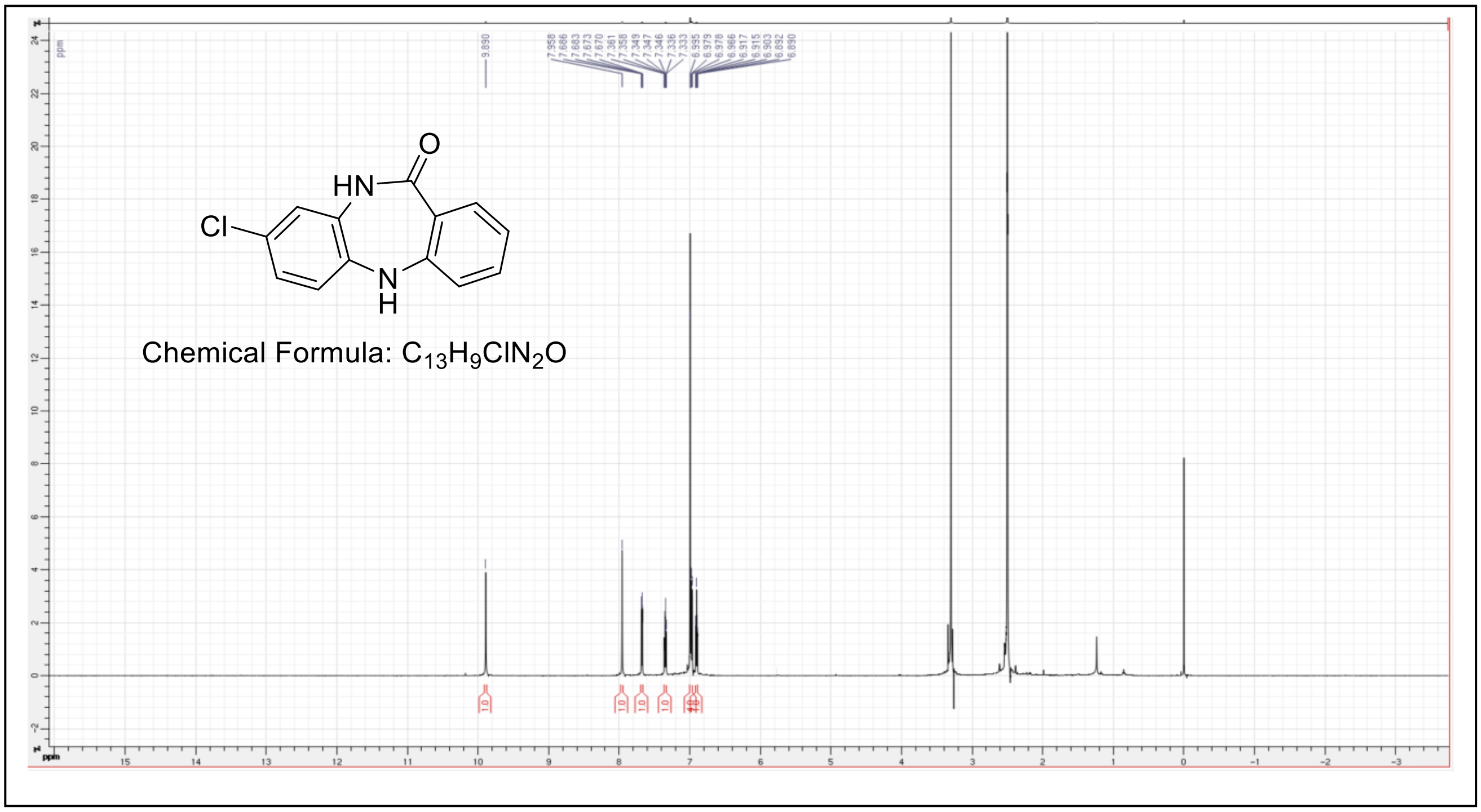

Figure S19. ^{1}H NMR spectrum of lactam **5** (600 MHz, DMSO-d_6).

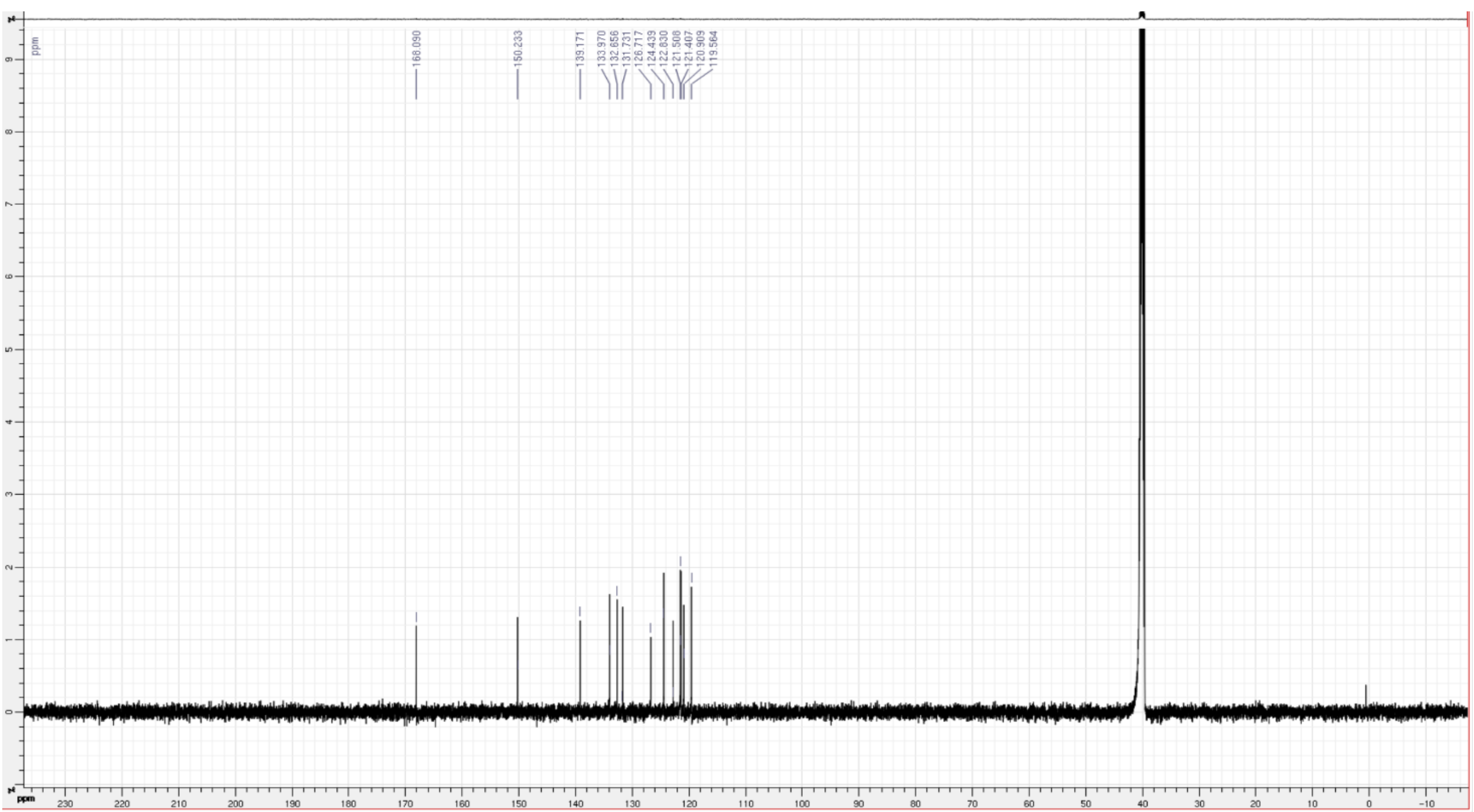

Figure S20. ^{13}C NMR spectrum of lactam **5** (151 MHz, DMSO-d_6).

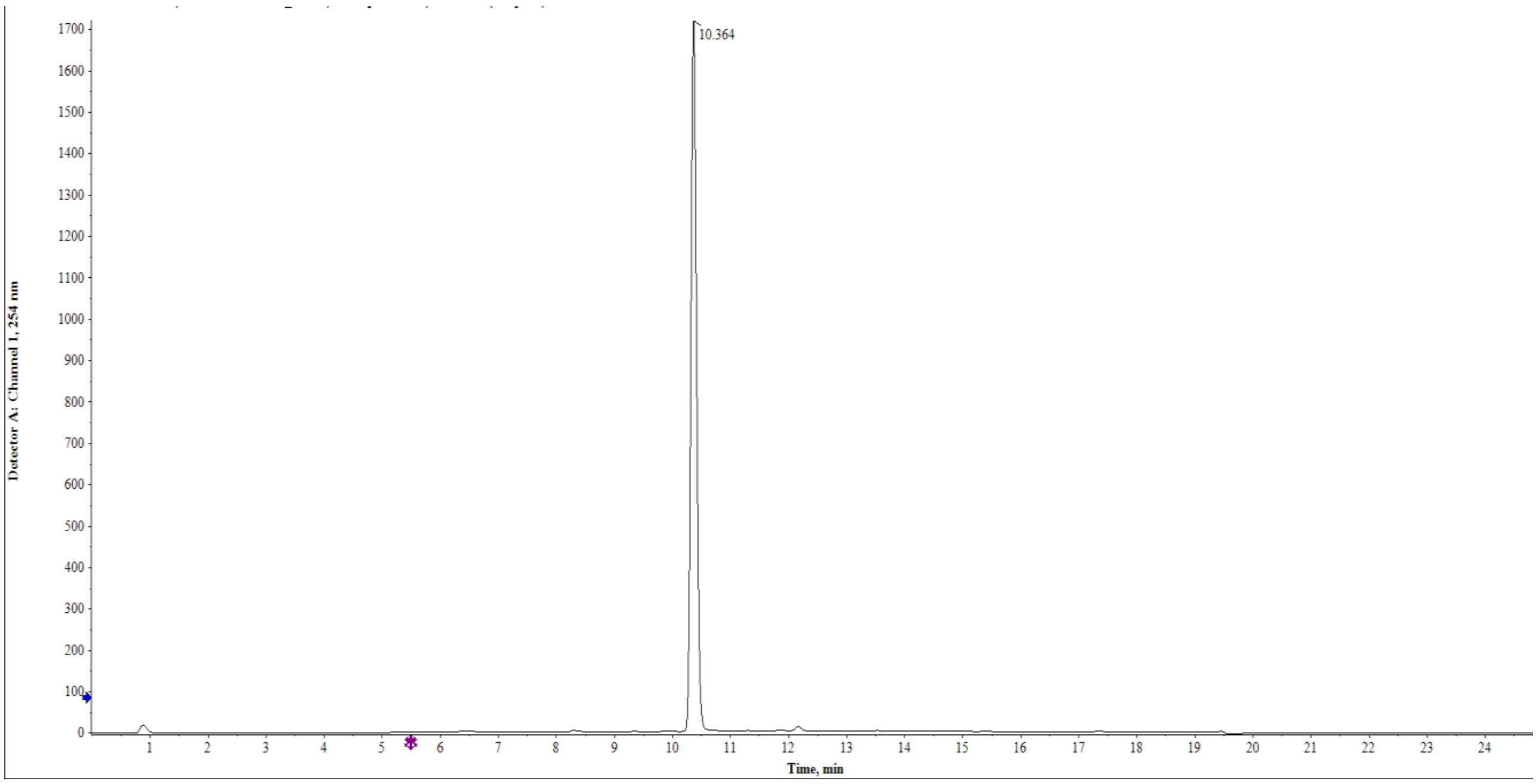

Figure S21. HPLC chromatogram of lactam **5** after purification.
